# Identification and Characterization of Leaf-Inhabiting Fungi from *Castanea* Plantations in China

**DOI:** 10.3390/jof7010064

**Published:** 2021-01-18

**Authors:** Ning Jiang, Xinlei Fan, Chengming Tian

**Affiliations:** The Key Laboratory for Silviculture and Conservation of Ministry of Education, Beijing Forestry University, Beijing 100083, China; 13146723574@163.com (N.J.); xinleifan@bjfu.edu.cn (X.F.)

**Keywords:** *Castanea henryi*, *C. mollissima*, Diaporthales, phylogeny, Sporocadaceae, taxonomy

## Abstract

Two *Castanea* plant species, *C. henryi* and *C. mollissima*, are cultivated in China to produce chestnut crops. Leaf spot diseases commonly occur in *Castanea* plantations, however, little is known about the fungal species associated with chestnut leaf spots. In this study, leaf samples of *C. henryi* and *C. mollissima* were collected from Beijing, Guizhou, Hunan, Sichuan and Yunnan Provinces, and leaf-inhabiting fungi were identified based on morphology and phylogeny. As a result, twenty-six fungal species were confirmed, including one new family, one new genus, and five new species. The new taxa are Pyrisporaceae fam. nov., *Pyrispora* gen. nov., *Aureobasidium castaneae* sp. nov., *Discosia castaneae* sp. nov., *Monochaetia castaneae* sp. nov., *Neopestalotiopsis sichuanensis* sp. nov. and *Pyrispora castaneae* sp. nov.

## 1. Introduction

*Castanea*, a plant genus well-known for edible chestnuts and hard timber, is distributed worldwide. The most four famous species are American chestnut (*C. dentata*), Chinese chestnut (*C. mollissima*), European chestnut (*C. sativa*) and Japanese chestnut (*C. crenata*). *C. mollissima* is widely cultivated as the crop in most provinces of China. In recent years, another *Castanea* species, *C. henryi*, was planted in Hunan Province of China to replace *C. mollissima* for its higher economic benefits.

In the long cultivation history of chestnut trees, fungal diseases have caused serious economic and ecological problems. Chestnut blight caused by *Cryphonectria parasitica* is the most notorious one, which devasted *Castanea dentata* forests in North America and weakened the other chestnut species in plantations [1,2,3]. Several emerging pathogens were recently reported in *Castanea sativa* from Australia and Europe, *viz*. *Gnomoniopsis smithogilvyi* (syn. *G. castaneae*) [4,5], *Phytophthora cinnamomi* [6], *Sirococcus castanea* [7].

In China, previous studies have revealed a high fungal diversity associated with chestnut branch cankers. For example, three *Coryneum* species [8], two *Cryphonectria* species [2], six *Cytospora* species and seven *Dendrostoma* species were described from the cankered branches [9,10]. In addition, some cryptic species were discovered, such as *Aurantiosacculus castaneae* [2], *Endothia chinensis* [2], *Melanops castaneicola* and *Neopseudomelanconis castaneae* [11,12]. Due to the high fungal diversity on chestnut branches, we collected leaf samples from two chestnut species, *Castanea henryi* and *C. mollissima* in China. In the present study, fungal taxa associated with the symptomatic leaves were identified based on morphological and molecular approaches, which is a fundamental task for the subsequent research on chestnut leaf diseases.

## 2. Materials and Methods

### 2.1. Field Sampling and Isolation

**Figure 1:**
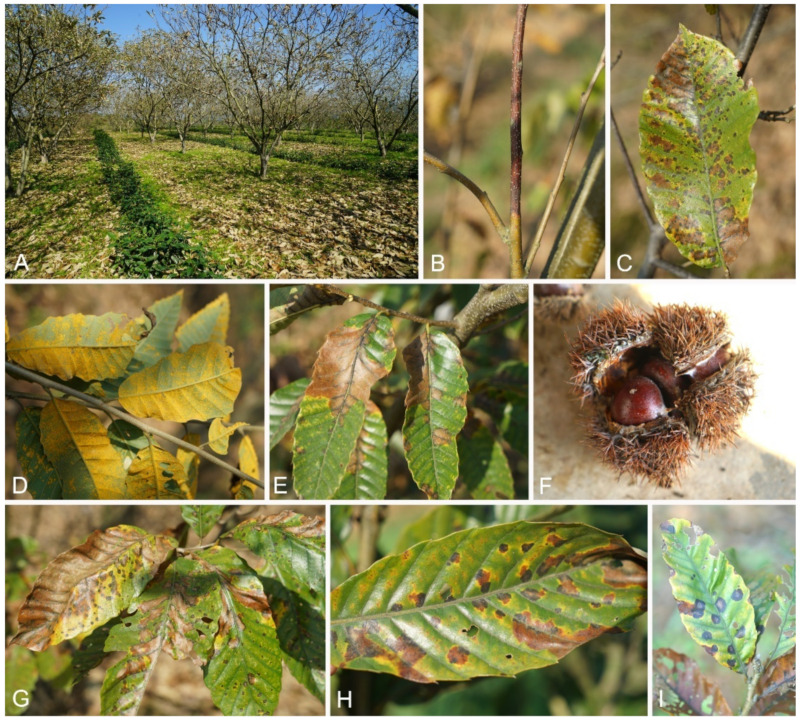
Field Sampling. (**A**) A *Castanea* plantation; (**B**) Symptoms of twig canker; (**C**–**E**,**G**–**I**) Symptoms of leaf diseases; (**F**) Chestnuts.

From 2017 to 2020, we investigated *Castanea* plantations of *C. henryi* and *C. mollissima* in the Beijing, Guizhou, Hunan, Sichuan and Yunnan Provinces of China. The disease symptoms were recorded (Figure 1), and 176 fresh leaf samples were collected and packed in sealed plastic bags. These leaf samples were transported to the laboratory for fungal isolation within ten days.

Fresh isolates were acquired by removing spore masses in fruiting bodies or surface-sterilized leaf tissues onto the surfaces of potato dextrose agar (PDA; 200 g potatoes, 20 g dextrose, 20 g agar per L) using axenic syringe needles. Then plates were incubated in the dark at 25 °C until germination. Hyphal tips were cut and transported to new PDA plates, and incubated in the dark at 25 °C.

### 2.2. Morphological Identification and Characterization

Fungal species on Chinese chestnut leaves were initially observed based on ascomata and conidiomata formed on the leaf surface or PDA, under a dissecting stereomicroscope (AZ100, Nikon, Tokyo, Japan), then asci, ascospores, conidiogenous cells and conidia were photographed using a Leica compound microscope (DM 2500, Leica, Wetzlar, Germany). Cultural characteristics of isolates incubated on PDA in the dark at 25 °C were recorded.

### 2.3. DNA Extraction, Sequencing and Phylogenetic Analysis

Genomic DNA was extracted from mycelium grown on PDA using a CTAB (cetyltrimethylammonium bromide) method [13]. Then PCR (polymerase chain reaction) was conducted for each genus using selected genes and primers (Table 1). The PCR conditions were set as follows: an initial denaturation step of 5 min at 94 °C followed by 35 cycles of 30 s at 94 °C, 50 s at 52 °C (ITS, LSU) or 54 °C (*act*, *cal*, *chs-1*, *gapdh*, *his3*, *rpb2*, *tub2*, *tef1*) and 1 min at 72 °C, and a final elongation step of 7 min at 72 °C. The PCR amplification products were sequenced using an ABI PRISM^®^ 3730XL DNA Analyzer with BigDye^®^ Terminater Kit v.3.1 (Invitrogen) at the Shanghai Invitrogen Biological Technology Company Limited (Beijing, China).

The sequences obtained in this study (Table 2) were supplemented with the additional sequences retrieved from GenBank. The sequences were aligned and checked manually using MEGA6. Ambiguous regions were excluded from the analyses and gaps were treated as missing data. Phylogenetic analyses were carried out with maximum likelihood analysis (ML), which was performed at the CIPRES web portal [14]. Bayesian inference analysis (BI) was performed in MrBayes v. 3.2.0 [15]. Phylogenetic trees were viewed in FigTree v1.4. The names of the isolates from present study are marked in blue in the trees. Maximum likelihood bootstrap support values ≥50% (BT) and Bayesian posterior probabilities ≥0.90 (PP) are given at the nodes respectively.

## 3. Results

A total of 26 species were identified, which belonging to two classes, six orders, 13 families and 18 genera in Ascomycota (Table 3). The seven new taxa are Pyrisporaceae fam. nov., *Pyrispora* gen. nov., *Aureobasidium castaneae* sp. nov., *Discosia castaneae* sp. nov., *Monochaetia castaneae* sp. nov., *Neopestalotiopsis sichuanensis* sp. nov. and *Pyrispora castaneae* sp. nov.

**Table 3:** Fungal species from *Castanea* leaves.

Phylum	Class	Order	Family	Species	Phogeny	Morphology
Ascomycota	Dothideomycetes	Botryosphaeriales	Aplosporellaceae	*Aplosporella prunicola*	Figure A1	NA
			Botryosphaeriaceae	*Botryosphaeria dothidea*	Figure A2	Figure 2
			Phyllostictaceae	*Phyllosticta capitalensis*	Figure A3	Figure 3 and Figure 4
		Dothideales	Saccotheciaceae	*Aureobasidium castaneae*	Figure 5	Figure 6
		Pleosporales	Didymellaceae	*Didymella coffeae-arabicae*	Figure A4	Figure 7
	Sordariomycetes	Amphisphaeriales	Apiosporaceae	*Arthrinium arundinis*	Figure A5	NA
			Sporocadaceae	*Bartalinia pini*	Figure A6	Figure 8
				*Discosia castaneae*	Figure 9	Figure 10
				*Monochaetia castaneae*	Figure 11	Figure 12
				*Neopestalotiopsis asiatica*	Figure 13	Figure 14
				*Neopestalotiopsis brasiliensis*	Figure 13	Figure 15
				*Neopestalotiopsis sichuanensis*	Figure 13	Figure 16
				*Neopestalotiopsis* sp.1	Figure 13	Figure 17
				*Neopestalotiopsis* sp.2	Figure 13	Figure 18
				*Pestalotiopsis kenyana*	Figure A7	Figure 19
				*Robillarda sessilis*	Figure A8	Figure 20
		Diaporthales	Diaporthaceae	*Diaporthe lithocarpi*	Figure A9	Figure 21
			Gnomoniaceae	*Gnomoniopsis daii*	Figure A10	Figure 22
			Pyrisporaceae	*Pyrispora castaneae*	Figure 23	Figure 24 and Figure 25
			Schizoparmaceae	*Coniella castaneicola*	Figure A11	Figure 26
			Tubakiaceae	*Tubakia dryinoides*	Figure A12	Figure 27
		Glomerellales	Glomerellaceae	*Colletotrichum fructicola*	Figure A13	NA
				*Colletotrichum henanense*	Figure A13	NA
				*Colletotrichum jiangxiense*	Figure A13	NA
				*Colletotrichum karsti*	Figure A14	NA
				*Colletotrichum nymphaeae*	Figure A15	NA

### 3.1. Aplosporella *Speg.*

*Aplosporella prunicola* Damm & Crous, Fungal Divers. 27(1): 39 (2007)

Endophytic or pathogenic on *Castanea mollissima* leaves. Sexual morph: Undetermined. Asexual morph: Undetermined.

Culture characteristics—Colonies on PDA reaching up to 60 mm in 5 days, flat with undulate edge, olivaceous to grey-olivaceous, aerial mycelium appressed, floccose, white to smoke-grey.

Material examined—CHINA, Sichuan Province, Yaan City, Shimian County, 29°13′31″ N, 102°21′27″ E, alt. 978 m, on leaf spots of *Castanea mollissima*, 10 September 2020, N. Jiang (living culture, CFCC 54334 = SM18B); *ibid*. (living culture, SM18B-1).

**Figure A1:**
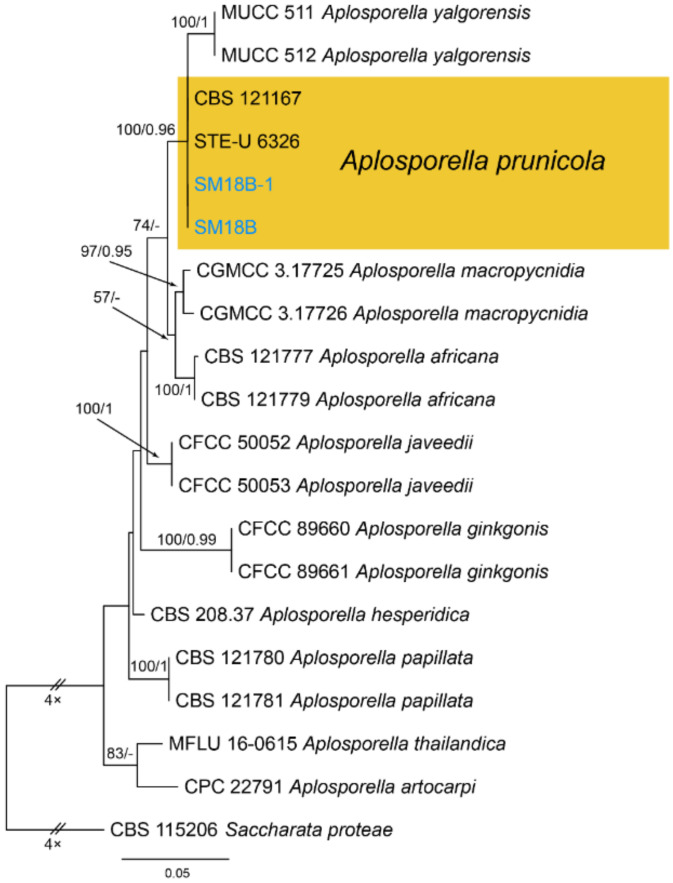
Phylogram generated from RAxML analysis based on combined ITS and *tef1* sequence data of *Aplosporella* isolates. The tree was rooted to *Saccharata proteae* (CBS 115206). The scale bar indicates 0.05 nucleotide changes per site. Isolates from this study are marked in blue, and the identified species is marked in yellow.

Notes—*Aplosporella prunicola* was initially recorded from *Prunus persica* var. *nucipersica* in South Africa [23]. Two new isolates from diseased chestnut leaves in present study are phylogenetically close to *Aplosporella prunicola* (Figure A1) and share similar culture characteristics on PDA. This is the first report of this fungus on the host *Castanea mollissima*, and in the Country China.

### 3.2. Botryosphaeria *Ces. & De Not.*

**Figure 2:**
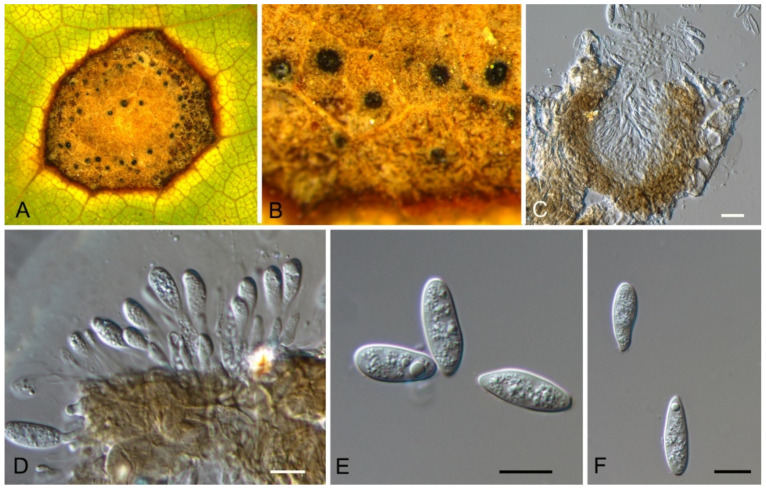
*Botryosphaeria dothidea* (BJFC-S1797). (**A**,**B**) Conidiomata on the diseased leaves of *Castanea mollissima*; (**C**) Section through the pycnidium; (**D**) Conidiogenous cells giving rise to conidia; (**E**,**F**) Conidia. Scale bars: C = 15 μm; C–F = 10 μm.

*Botryosphaeria dothidea* Ces. & De Not., Comm. Soc. crittog. Ital. 1(fasc. 4): 212 (1863) (Figure 2)

Pathogenic on *Castanea henryi* and *C. mollissima* leaves. Sexual morph: Undetermined. Asexual morph: Conidiomata 100–250 μm diam., pycnidial, aggregated, globose, black. Conidiophores absent. Conidiogenous cells 3–11 × 1.5–3 μm, holoblastic, discrete, hyaline, cylindrical to lageniform, phialidic with periclinal thickening. Conidia (18.3–)20–24.7(–26.4) × (6.3–)7.2–8.4(–8.7) μm ($\bar{x}$ = 22.3 × 7.8 μm, *n* = 50), L/W = (2.3–)2.4–3.4(–3.9) ($\bar{x}$ = 2.9, *n* = 50), hyaline, thin-walled, smooth with granular contents, unicellular, aseptate, ellipsoid to fusoid, base subtruncate to bluntly rounded.

Culture characteristics—Colonies on PDA reaching up to 60 mm in 4 days, initially white with fluffy, aerial mycelium, becoming black on the surface after 5 days; reverse side of the colonies dark-brown.

Material examined—CHINA, Hunan Province, Changsha City, Changsha County, Jinjing Town, 28°58′52″ N, 113°34′38″ E, alt. 63 m, on leaf spots of *Castanea henryi*, 10 November 2020, C.M. Tian & N. Jiang (BJFC-S1823; living culture, JJ 2B); *ibid*. (living cultures, CFCC 54576 = JJ12, JJ14, JJ27-1); Hunan Province, Shaoshan City, Yintian Town, 27°52′04″ N, 112°35′03″ E, alt. 73 m, on leaf spots of *Castanea mollissima*, 9 November 2020, C.M. Tian & N. Jiang (living culture, CFCC 54585 = SS9-1); Beijing City, Haidian District, 40°00′30″ N, 116°20′26″ E, alt. 23 m, on leaf spots of *Castanea mollissima*, 28 August 2020, N. Jiang (BJFC-S1797).

**Figure A2:**
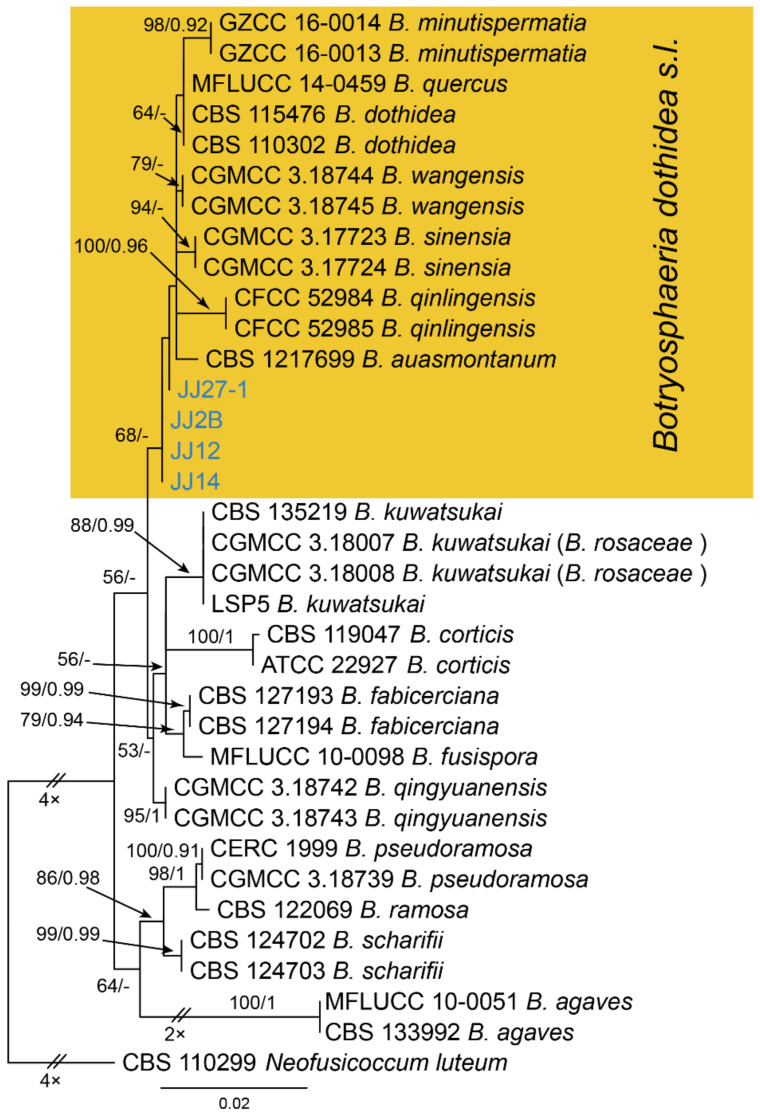
Phylogram generated from RAxML analysis based on combined ITS, *tef1* and *tub2* sequence data of Botryosphaeria isolates. The tree was rooted to *Neofusicoccum luteum* (CBS 110229). The scale bar indicates 0.02 nucleotide changes per site. Isolates from this study are marked in blue, and the identified species is marked in yellow.

Notes—The genus *Botryosphaeria* is presently not well separated phylogenetically [24,25,26]. In the phylogenetic inference of ITS, *tef1* and *tub2* sequence data (Figure A2), four new isolates from this study are close to *B. auasmontanum*, *B. dothidea*, *B. minutispermatia*, *B. qinlingensis*, *B. sinensis* and *B. wangensis*. Phylogenic analyses based on more collections of this genus are necessary to clarify species concept in the future. In present study, we provide the morphology and phylogeny of samples from *Castanea* leaves, and identified these isolates as *Botryosphaeria dothidea s. l.*

### 3.3. Phyllosticta *Pers.*

**Figure 3:**
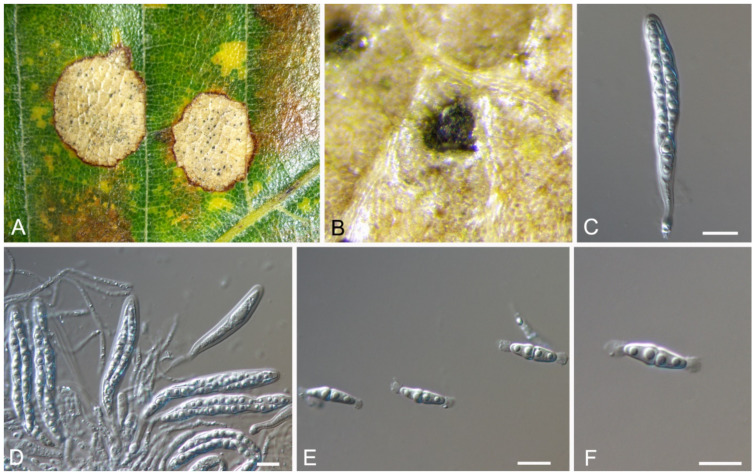
*Phyllosticta capitalensis* (BJFC-S1821). (**A**,**B**) Ascostromata on the diseased leaves of *Castanea mollissima*; (**C**,**D**) Asci; (**E**,**F**) Ascospores. Scale bars: C–F = 10 μm.

**Figure 4:**
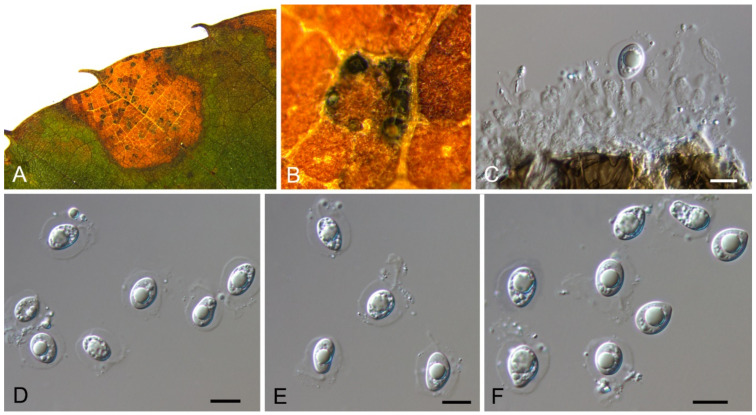
*Phyllosticta capitalensis* (BJFC-S1820). (**A**,**B**) Conidiomata on the diseased leaves of *Castanea mollissima*; (**C**) Conidiogenous cells giving rise to conidia; (**D**–**F**) Conidia. Scale bars: C–F = 10 μm.

*Phyllostictacapitalensis* Henn., Hedwigia 48: 13 (1908) (Figure 3 and Figure 4)

Pathogenic on *Castanea henryi* and *C. mollissima* leaves. Sexual morph: Ascomata 80–200 μm diam., globose to pyriform, black. Asci 62–85 × 8–12.5 μm, bitunicate, clavate to broadly fusoid-ellipsoid. Ascospores (13.2–)14.6–16.2(–17.4) × (3.7–)4.1–5.2(–6.8) μm ($\bar{x}\;$= 15.1 × 4.5 μm, *n* = 30), L/W = (2.8–)2.9–4(–4.1) ($\bar{x}$ = 3.5, *n* = 30), bi-seriate, hyaline, smooth, 1-septate or aseptate, guttulate, straight and slightly curved, widest in the middle, limoniform with obtuse ends, with distinct hyaline gelatinous caps at both ends, 3–5 × 1.5–3 μm. Asexual morph: Conidiomata 100–250 μm diam., pycnidial, scattered or aggregated, globose to subglobose, black. Pycnidial wall of several layers, composed of cells of textura angularis, thick, inner wall of hyaline textura angularis cells. Conidiophores reduced to conidiogenous cells. Conidiogenous cells 3–12 × 2.5–5.5 μm, terminal, subcylindrical to ampulliform to doliiform, hyaline, smooth, proliferating several times percurrently near apex. Conidia (9–)10–11.8(–12.4) × (6.8–)7.4–8.3(–8.6) μm ($\bar{x}\;$= 10.9 × 7.8 μm, *n* = 50), L/W = (1.1–)1.3–1.5(–1.6) ($\bar{x}$ = 1.4, *n* = 50), solitary, hyaline, aseptate, thin and smooth walled, with a single large central guttulate, obovoid, tapering towards a narrow truncate base, enclosed in a persistent mucoid sheath, 2–7 μm thick.

Culture characteristics—Colonies on PDA reaching up to 40 mm in 10 days, flat, initially white-grey mycelium, gradually becoming greenish to dark brown, with white hyphae at the margin; reverse black.

Material examined—CHINA, Hunan Province, Changsha City, Changsha County, Jinjing Town, 28°58′52″ N, 113°34′38″ E, alt. 63 m, on leaf spots of *Castanea henryi*, 10 November 2020, C.M. Tian & N. Jiang (BJFC-S1821; living culture, JJ4); *ibid*. (BJFC-S1822; living culture, CFCC 54576 = JJ11); *ibid*. (living cultures, CFCC 54577 = JJ16, JJ20); Hunan Province, Shaoshan City, Yintian Town, 27°52′04″ N, 112°35′03″ E, alt. 73 m, on leaf spots of *Castanea mollissima*, 9 November 2020, C.M. Tian & N. Jiang (BJFC-S1820; living culture, SS5); *ibid*. (living cultures, SS10, SS13, SS15, SS16-1, SS16-2); Hunan Province, Xiangtan City, 27°48′51″ N, 112°71′42″ E, alt. 85 m, on leaf spots of *Castanea mollissima*, 9 November 2020, N. Jiang (BJFC-S1819; living culture, XT2); *ibid*. (living cultures, XT10, XT11, XT16-1, XT16-2, XT17); Yunnan Province, Zhaotong City, Zhenxiong County, 27°43′28″ N, 105°10′35″ E, alt. 1280 m, on leaf spots of *Castanea mollissima*, 5 September 2020, N. Jiang (living cultures, ZX6, ZX11-1); Guizhou Province, Zunyi City, Goujiang Town, 27°24′49″ N, 106°52′49″ E, alt. 1064 m, on leaf spots of *Castanea mollissima*, 7 September 2020, N. Jiang (living culture, ZY6-1).

**Figure A3:**
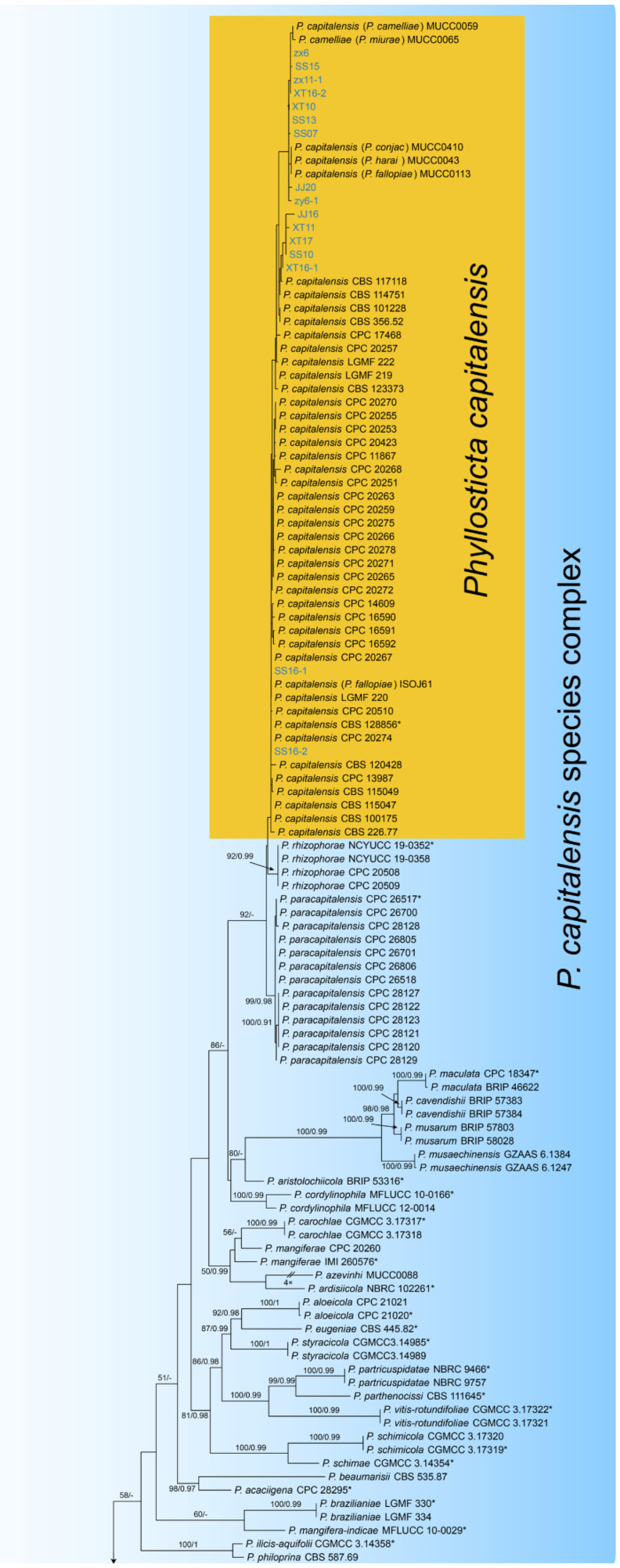

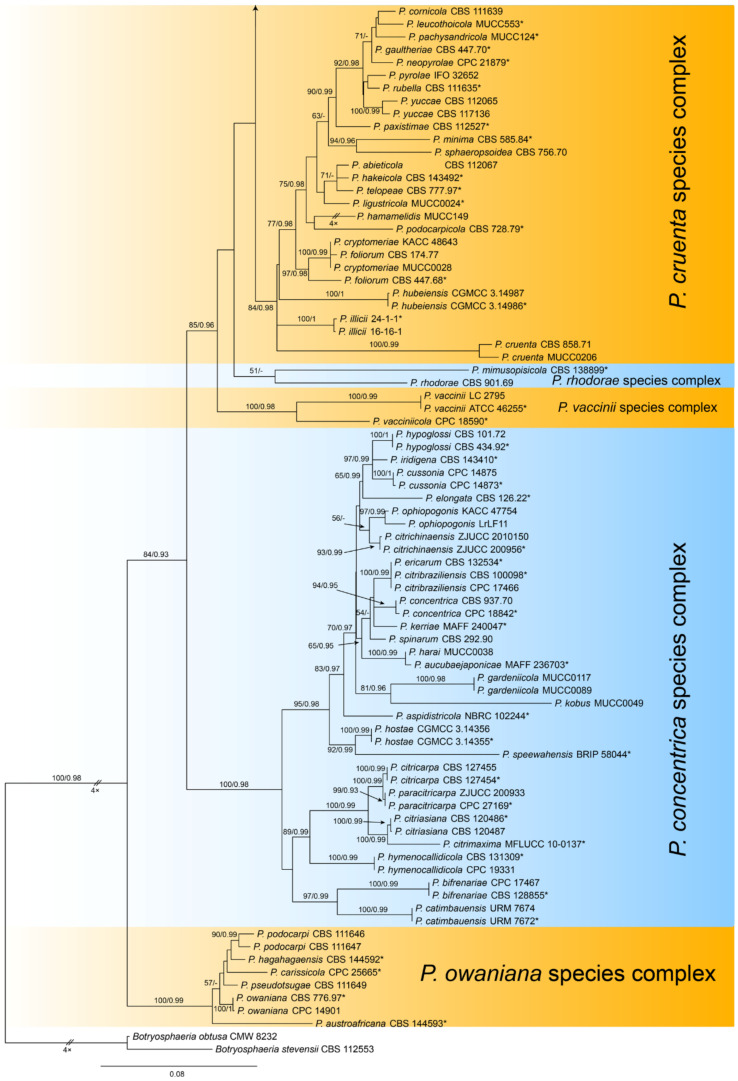
Phylogram generated from RAxML analysis based on combined ITS, LSU, *act*, *gapdh* and *tef1* sequence data of *Phyllosticta* isolates. The tree was rooted to *Botryosphaeria obtusa* (CMW 8232) and *B. stevensii* (CBS 112553). The scale bar indicates 0.08 nucleotide changes per site. Isolates from this study are highlighted, and ex-type strains are marked with *.

Notes—The genus *Phyllosticta* is a worldwide genus of pathogens, endophytes and saprobes, which was separated into six species complexes [27]. In the present study, we observed the sexual and asexual morphs on *Castanea* leaves, and identified them as *Phyllosticta capitalensis s. s.* based on the phylogenetic inference of ITS, LSU, *act*, *gapdh* and *tef1* sequence data (Figure A3).

### 3.4. Aureobasidium *Viala & G. Boyer*

*Aureobasidium castaneae* C.M. Tian & N. Jiang, sp. nov. (Figure 6)

MycoBank: MB 838314

Etymology—named after the host genus, *Castanea*.

Holotype—BJFC-C007

Endophytic or pathogenic on *Castanea henryi* leaves. Sexual morph: Undetermined. Asexual morph was observed on PDA: Mycelium immersed, no aerial mycelium. Hyphae 3–7 μm wide, smooth, thin-walled, septate, hyaline or brown. Conidiogenous cells undifferentiated, hyaline or brown, intercalary or rarely terminal. Conidia (5.9–)7.1–10.6(–11.8) × (2.7–)2.7–4.7(–6.2) μm ($\bar{x}$ = 8.9 × 3.7 μm, *n* = 50), L/W = (1.8–)2.1–2.8(–3.1) ($\bar{x}$ = 2.4, *n* = 50), hyaline or brown based on the color of conidiogenous cells, variable in size, ellipsoidal, straight, rarely slightly curved, with rounded to subtruncate base and a flat basal hilum, thin-walled, with two or more guttules.

Culture characteristics—Colonies on PDA reaching up to 40 mm in 7 days, spreading, smooth, flat, rapidly turning to olivaceous black, with dark green, irregular margins, covered with slimy masses of conidia.

Material examined—CHINA, Hunan Province, Changsha City, Changsha County, Jinjing Town, 28°58′52″ N, 113°34′38″ E, alt. 63 m, on leaf spots of *Castanea henryi*, 10 November 2020, C.M. Tian & N. Jiang (BJFC-C007, holotype; ex-type living culture, CFCC 54591 = JJ 7-3).

**Figure 5:**
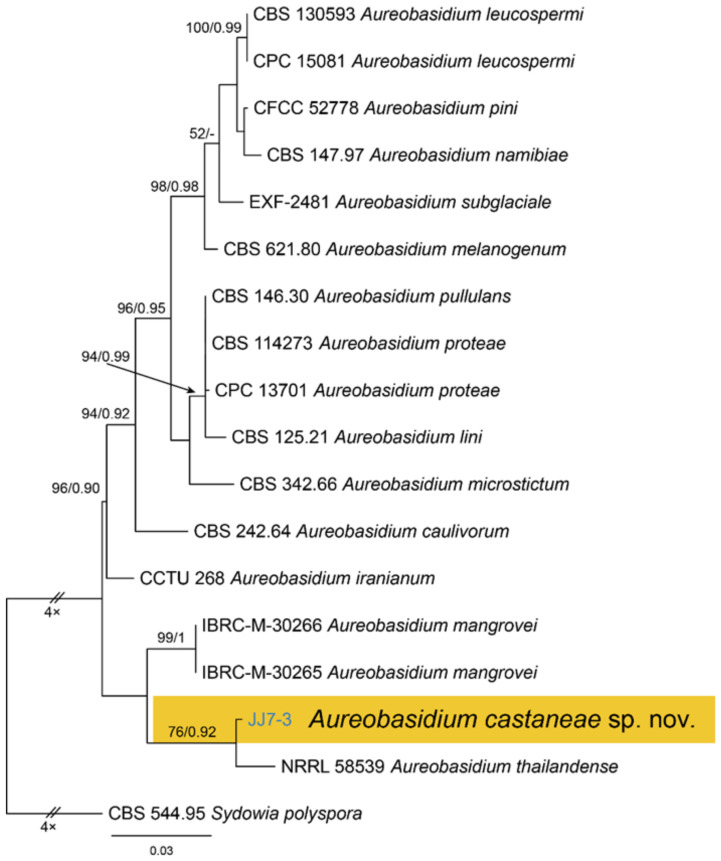
Phylogram generated from RAxML analysis based on combined ITS and LSU sequence data of *Aureobasidium* isolates. The tree was rooted to *Sydowia polyspora* (CBS 544.95). The scale bar indicates 0.03 nucleotide changes per site. Isolate from this study is marked in blue, and the identified species is marked in yellow.

**Figure 6:**
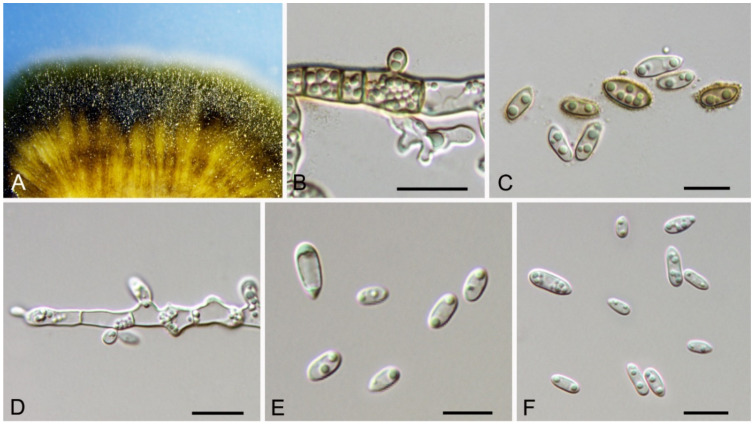
*Aureobasidium castaneae* (BJFC-C007, holotype). (**A**) Colony on PDA; (**B**,**D**) Conidiogenous cells and conidia; (**C**,**E**,**F**) Conidia. Scale bars: B–F = 10 μm.

Notes—*Aureobasidium* is a well-known genus comprising saprophytes, endophytes and pathogens on various substrates [28,29,30,31,32]. *A. castaneae* from *Castanea henryi* in this study is closely related to *A. thailandense* from unknown plants based on phylogenetic inference of ITS and LSU sequence data (Figure 5). However, *A. castaneae* can be easily distinguished from *A. thailandense* by conidial size (7.1–10.6 × 2.7–4.7 μm in *A. castaneae* vs. 3–10 × 5–12 μm in *A. thailandense*) [31].

### 3.5. Didymella *Sacc.*

**Figure 7:**
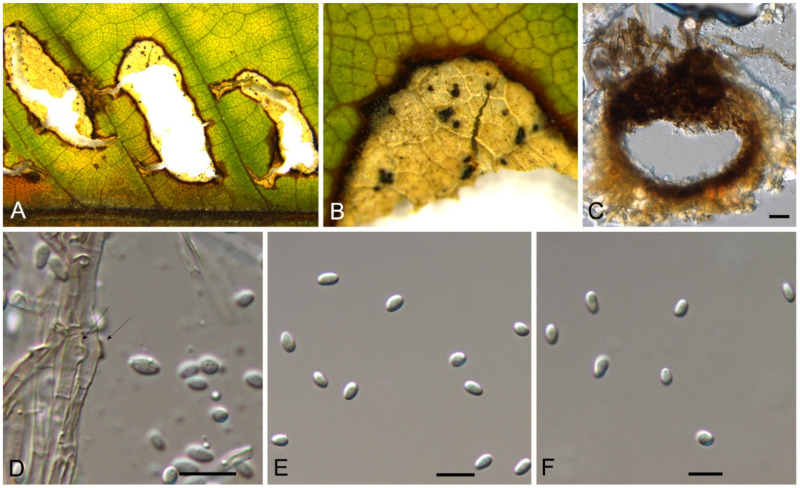
*Didymella coffeae-arabicae* (BJFC-S1792). (**A**,**B**) Conidiomata on the diseased leaves of *Castanea mollissima*; (**C**) Section through the pycnidium; (**D**) Conidiogenous cells giving rise to conidia (arrows); (**E**,**F**) Conidia. Scale bars: C–F = 10 μm.

*Didymella coffeae-arabicae* Qian Chen & L. Cai, Stud. Mycol. 82: 175 (2015) (Figure 7)

Pathogenic on *Castanea mollissima* leaves. Sexual morph: Undetermined. Asexual morph: Conidiomata 120–200 μm diam., 100–180 μm high, pycnidial, conspicuous, stromatic, scattered or aggregated, globose to subglobose, dark brown, with septate and dark hyphal outgrowths. Ostioles single, centric. Pycnidial wall pseudoparenchymatous, composed of isodiametric cells, 3–5 layers, 8–20 μm thick. Conidiogenous cells phialidic, hyaline, simple, smooth, flask-shaped to globose. Conidia (3.1–)3.7–4.8(–4.9) × (1.8–)2.1–2.7(–2.8) μm ($\bar{x}\;$= 4.3 × 2.4 μm, *n* = 50), L/W = (1.3–)1.5–2.2(–2.6) ($\bar{x}\;$= 1.8, *n* = 50), ellipsoidal to ovoid, thin-walled, smooth, hyaline, always aseptate, eguttulate or with several minute apolar guttules.

Culture characteristics—Colonies on PDA reaching up to 70 mm in 7 days, margin regular, covered by felty aerial mycelium, olivaceous, mouse grey towards periphery, reverse dark mouse grey.

Material examined—CHINA, Sichuan Province, Yaan City, Shimian County, 29°13′31″ N, 102°21′27″ E, alt. 978 m, on leaf spots of *Castanea mollissima*, 10 September 2020, N. Jiang (BJFC-S1792; living culture, CFCC 54343 = SM24); *ibid*. (living culture, SM24B).

**Figure A4:**
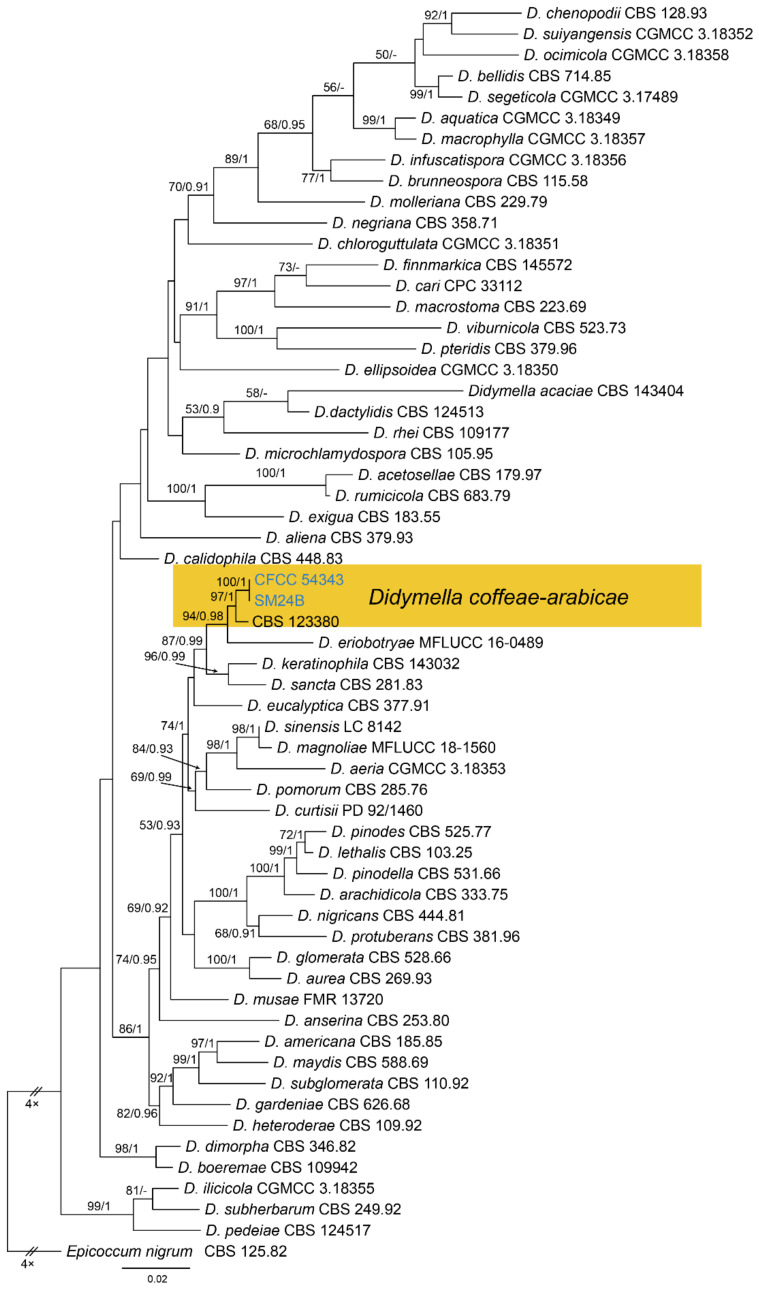
Phylogram generated from RAxML analysis based on combined ITS, LSU, *rpb2* and *tub2* sequence data of *Didymella* isolates. The tree was rooted to *Epicoccum nigrum* (CBS 125.82). The scale bar indicates 0.02 nucleotide changes per site. Isolates from this study are marked in blue, and the identified species is marked in yellow.

Notes—*Didymella* is a famous pathogenic genus mainly occurring on plant leaves [33,34,35,36,37]. Phoma-like species are difficult to identify to the genus and species level due to a lack of good characteristics, making molecular data are essential during species identification [34,35,37]. The new isolates (CFCC 54343 and SM24B) from *Castanea mollissima* grouped with *Didymella coffeae-arabicae* on *Coffea arabica* with high statistical support (Figure A4). Our collection also shared similar morphology in conidial size with *Didymella coffeae-arabicae* [33].

### 3.6. Arthrinium *Kunze*

*Arthrinium arundinis* (Corda) Dyko & B. Sutton, Mycotaxon 8(1): 119 (1979)

Endophytic or pathogenic on *Castanea mollissima* leaves. Sexual morph: Undetermined. Asexual morph was observed on PDA: Mycelium consisting of smooth, hyaline, branched, septate, 1.5–3 μm diam hyphae. Conidiogenous cells 5.5–15 × 2.5–3.5 μm, aggregated in clusters on hyphae, pale brown, smooth, ampulliform. Conidia 3–4 μm diam. ($\bar{x}\;$= 3.4 μm, *n* = 50), brown, smooth, globose, with pale equatorial slit.

Culture characteristics—Colonies on PDA reaching up to 70 mm in 7 days, flat, spreading, covered by iron-grey aerial mycelium, reverse grey.

Material examined—CHINA, Hunan Province, Xiangtan City, 27°48′51″ N, 112°71′42″ E, alt. 85 m, on leaf spots of *Castanea mollissima*, 9 November 2020, N. Jiang (living culture, XT18-1).

**Figure A5:**
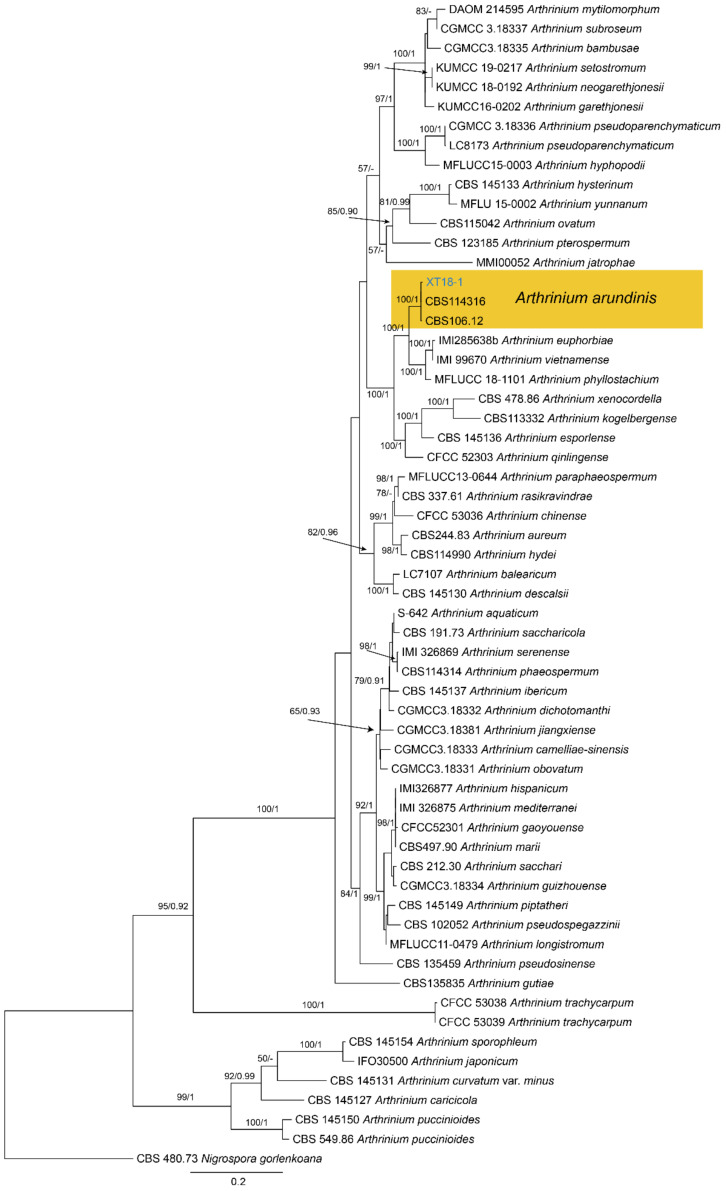
Phylogram generated from RAxML analysis based on combined ITS, *tef1* and *tub2* sequence data of *Arthrinium* isolates. The tree was rooted to *Nigrospora gorlenkoana* (CBS 480.73). The scale bar indicates 0.2 nucleotide changes per site. Isolate from this study is marked in blue, and the identified species is marked in yellow.

Notes—*Arthrinium* is a common genus on a wide range of substrates, including soil, plant debris, plants, lichens, marine algae, and human tissues [38,39,40,41,42]. The new isolate (XT18-1) from *Castanea mollissima* grouped with *A. arundinis* isolates with high statistical support (Figure A5). Therefore, we identify our new isolate as *A. arundinis* and *Castanea mollissima* as a new host record for the fungus.

### 3.7. Bartalinia *Tassi*

**Figure 8:**
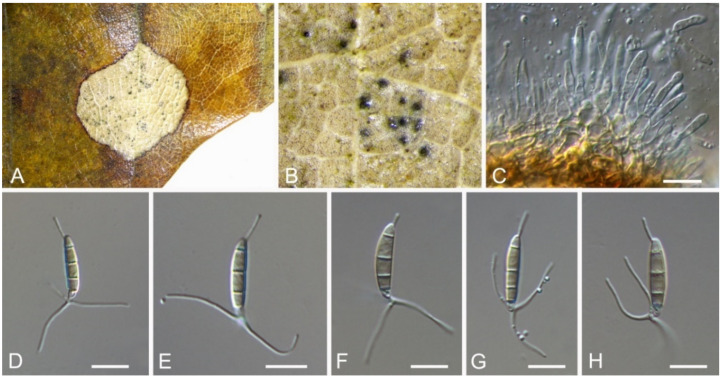
*Bartalinia pini* (BJFC-S1808). (**A**,**B**) Conidiomata on the diseased leaves of *Castanea henryi*; (**C**) Conidiogenous cells giving rise to conidia; (**D**–**H**) Conidia. Scale bars: C–F = 10 μm.

*Bartalinia pini* F. Liu, L. Cai & Crous, Stud. Mycol. 92: 309 (2019) (Figure 8)

Pathogenic on *Castanea henryi* leaves. Sexual morph: Undetermined. Asexual morph: Conidiomata 85–160 μm diam., 50–120 μm high, acervular, conspicuous, stromatic, scattered or aggregated, rounded, black. Conidiophores reduced to conidiogenous cells, septate, smooth, hyaline, invested in mucus. Conidiogenous cells 4.5–15 × 1.5–4 μm, annellidic, discrete, ampulliform. Conidia (18.2–)19–21.5(–22.1) × (3.4–)3.6–4.2(–4.5) μm ($\bar{x}\;$= 20.1 × 3.8 μm, *n* = 50), L/W = (4.5–)4.7–5.9(–6.2) ($\bar{x}\;$= 5.3, *n* = 50), cylindrical with acute or obtuse ends, straight or slightly curved, 4-septate, smooth; basal cell obconic with a truncate base, thin-walled, hyaline, 1.5–3.5 μm long; median cells 3, cylindrical, pale brown, each 3.5–7.5 μm long; apical cell conic with an acute apex, thin-walled, hyaline, 2–3.5 μm long; apical appendage with three branches, tubular, filiform, flexuous, divergent, 5–25 μm long; basal appendage tubular, unbranched, excentric, 3–8 μm long.

Culture characteristics—Colonies on PDA reaching up to 70 mm in 10 days, flat with entire edge, glaucous grey to grey.

Material examined—CHINA, Hunan Province, Changsha City, Changsha County, Jinjing Town, 28°58′52″ N, 113°34′38″ E, alt. 63 m, on leaf spots of *Castanea henryi*, 10 November 2020, C.M. Tian & N. Jiang (BJFC-S1808; living culture, CFCC 54574 = JJ4).

**Figure A6:**
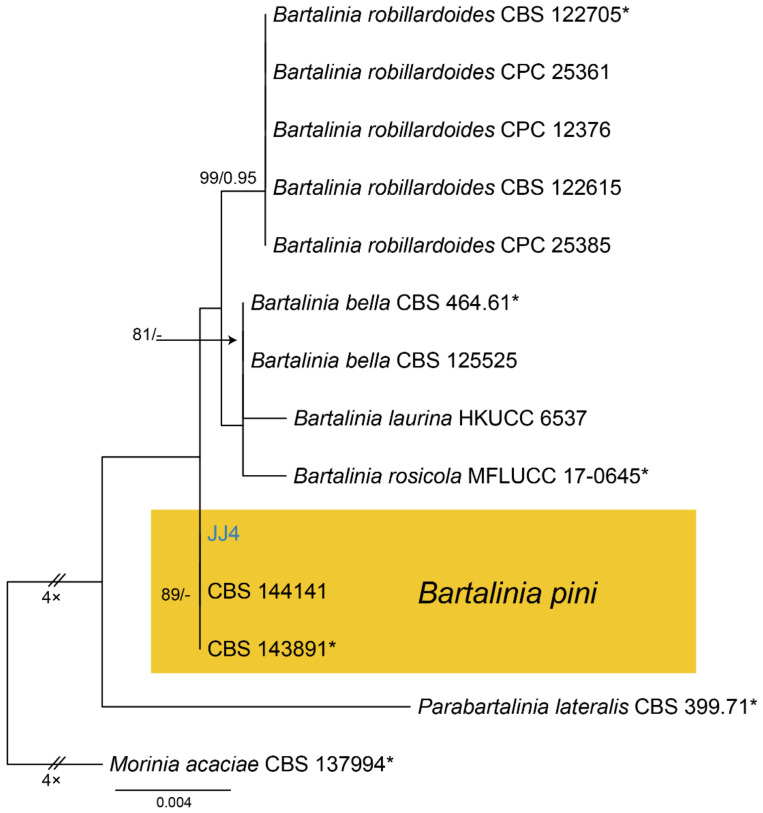
Phylogram generated from RAxML analysis based on combined ITS and LSU sequence data of *Bartalinia* isolates. Table 137994. The scale bar indicates 0.004 nucleotide changes per site. Isolate from this study is marked in blue, ex-type strains are marked with *, and the identified species is marked in yellow.

Notes—*Bartalinia* is morphologically distinct from the other Sporocadaceous genera by its conidial characters [43]. The new isolate (CFCC 54574) from *Castanea henryi* grouped with *B. pini* isolates from *Pinus patula* (Pinaceae) needles and leaves of *Acacia koa* (Fabaceae) (Figure A6) and overlapped in morphology with *B. pini* [43]. Therefore, we identify our new isolate as *B. pini*, China as a new geographical record and *Castanea henryi* as a new host record for the fungus.

### 3.8. Discosia *Lib. ex Durieu & Mont.*

*Discosia castaneae* C.M. Tian & N. Jiang, sp. nov. (Figure 10)

MycoBank: MB837794

Etymology—named for the host genus, *Castanea*.

Holotype—BJFC-S1805

Pathogenic on *Castanea mollissima* leaves. Sexual morph: Undetermined. Asexual morph: Conidiomata 100–170 μm diam., 20–40 μm high, acervular, conspicuous, stromatic, scattered or aggregated, rounded, black, glabrous, epidermal. Conidiophores absent. Conidiogenous cells 3–7 × 1–3 μm, holoblastic to phialidic, ampulliform, integrated, hyaline, smooth-walled. Conidia (14.1–)15.4–17(–18.7) × (2.9–)3.1–3.8(–3.9) μm ($\bar{x}\;$= 16.2 × 3.5 μm, *n* = 50), L/W = (3.9–)4.2–5.3(–5.5) ($\bar{x}\;$= 4.8, *n* = 50), cylindrical to allantoid, initially hyaline, becoming pale brown at maturity, smooth-walled, guttulate, 3-euseptate, slightly constricted at septa, thin-walled; with basal cell obconic, slightly truncate at the base and appendaged; 2 median cells subcylindrical, with second cell from the base 5–8 μm long and third cell 3–6 μm long; apical cell subconical with a obtuse apex; apical and basal cells each with a single, simple, unbranched, filamentous appendage at the ends, apical appendage 5–12 μm and basal appendage 5–10 μm.

Culture characteristics—Colonies on PDA reaching up to 50 mm in 10 days, flat with entire edge, forming concentric circles, olivaceous black, sterile.

Material examined—CHINA, Beijing City, Miyun District, Xinchengzi Town, Potou Village, 40°60′28″ N, 117°36′02″ E, alt. 65 m, on leaf spots of *Castanea mollissima*, 29 October 2017, C.M. Tian & N. Jiang (BJFC-S1805, holotype; ex-type living cultures, CFCC 54088 = CML1, CML2). Yunnan Province, Zhaotong City, Zhenxiong County, 27°43′28″ N, 105°10′35″ E, alt. 1280 m, on leaf spots of *Castanea mollissima*, 5 September 2020, N. Jiang (living culture, CFCC 54352 = ZX22A). Hunan Province, Shaoshan City, Yintian Town, 27°52′04″ N, 112°35′03″ E, alt. 73 m, on leaf spots of *Castanea mollissima*, 9 November 2020, C.M. Tian & N. Jiang (BJFC-S1806, living culture, CFCC 54584 = SS3).

**Figure 9:**
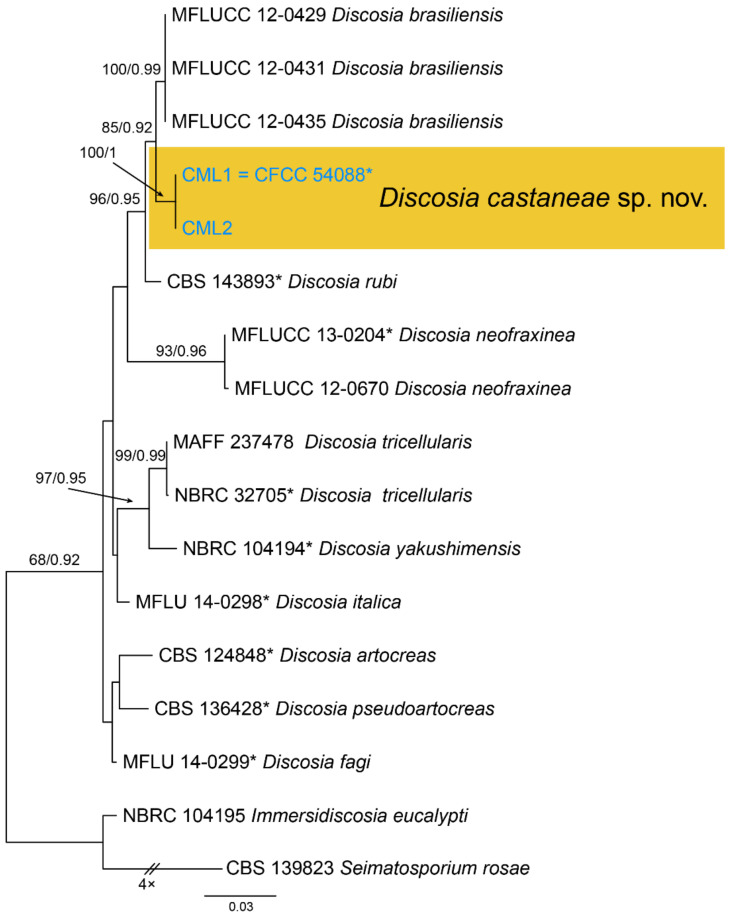
Phylogram generated from RAxML analysis based on combined ITS, LSU and *tub2* sequence data of *Discosia* isolates. The tree was rooted to *Immersidiscosia eucalypti* (CBS 544.95) and *Seimatosporium rosae* (CBS 139823). The scale bar indicates 0.03 nucleotide changes per site. Isolates from this study are marked in blue, ex-type strains are marked with *, and the identified species is marked in yellow.

**Figure 10:**
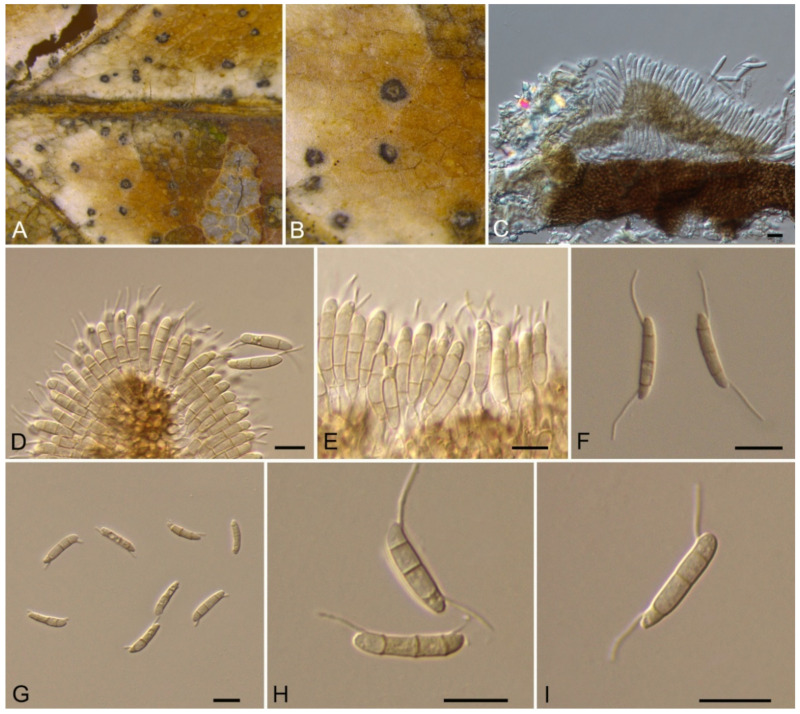
*Discosia castaneae* (BJFC-S1805). (**A**,**B**) Conidiomata on the diseased leaves of *Castanea mollissima*; (**C**) Section through the conidioma; (**D**,**E**) Conidiogenous cells giving rise to conidia; (**F**–**I**) Conidia. Scale bars: C–I = 10 μm.

Notes—*Discosia* was recorded on leaf spots of Chinese chestnut leaves in the monograph of chestnut diseases [44]. However, the fungus was not identified to the species level. In present study, we collected *Discosia* samples from Beijing (North China), Hunan and Yunnan (South China), and found it different from any known species [43,45,46]. From the phylogram (Figure 9), *Discosia brasiliensis* was the closest species to *D. castaneae*, but they could be distinguished by their conidial width (3.5–4.5 μm in *D. castaneae* vs. 2–3 μm in *D. brasiliensis*) [46].

### 3.9. Monochaetia *(Sacc.) Allesch.*

*Monochaetia castaneae* C.M. Tian & N. Jiang, sp. nov. (Figure 12)

MycoBank: MB837793

Etymology—named for the host genus, *Castanea*.

Holotype—BJFC-S1807

Pathogenic on *Castanea mollissima* leaves. Sexual morph: Undetermined. Asexual morph: Conidiomata 120–230 μm diam., 20–50 μm high, acervular, conspicuous, scattered or aggregated, rounded, black. Conidiophores cylindrical, hyaline, smooth-walled. Conidiogenous cells 12–20 × 1.5–2.5 μm, phialidic, ampulliform, discrete, hyaline, smooth-walled. Conidia (18.8–)20–24(–27.3) × (4.7–)5.4–6.2(–6.6) μm ($\bar{x}\;$= 22 × 5.8 μm, *n* = 50), L/W = (3.1–)3.2–4.5(–5.8) ($\bar{x}\;$= 3.8, *n* = 50), fusiform, tapering at both ends, 4-septate; basal cell 2.5–4.0 μm long, conic, hyaline and smooth-walled; three median cells each 3.5–5.5 μm long, doliiform, light brown, rough-walled; apical cell 3.0–4.5 μm long, conic, hyaline and smooth-walled; apical appendage 17.5–35 μm long, single, tubular, filiform; basal appendage 10–20 μm long, single, central, tubular, filiform.

Culture characteristics—Colonies on PDA reaching up to 40 mm in 15 days, flat, spreading, with sparse aerial mycelium and smooth, lobate margin, cinnamon, reverse isabelline. Fruiting bodies were observed after 20 days.

Material examined—CHINA, Sichuan Province, Yaan City, Shimian County, 29°13′31″ N, 102°21′27″ E, alt. 978 m, on leaf spots of *Castanea mollissima*, 10 September 2020, N. Jiang (BJFC-S1807, holotype; ex-type living cultures, CFCC 54354 = SM9-1, SM9-2).

**Figure 11:**
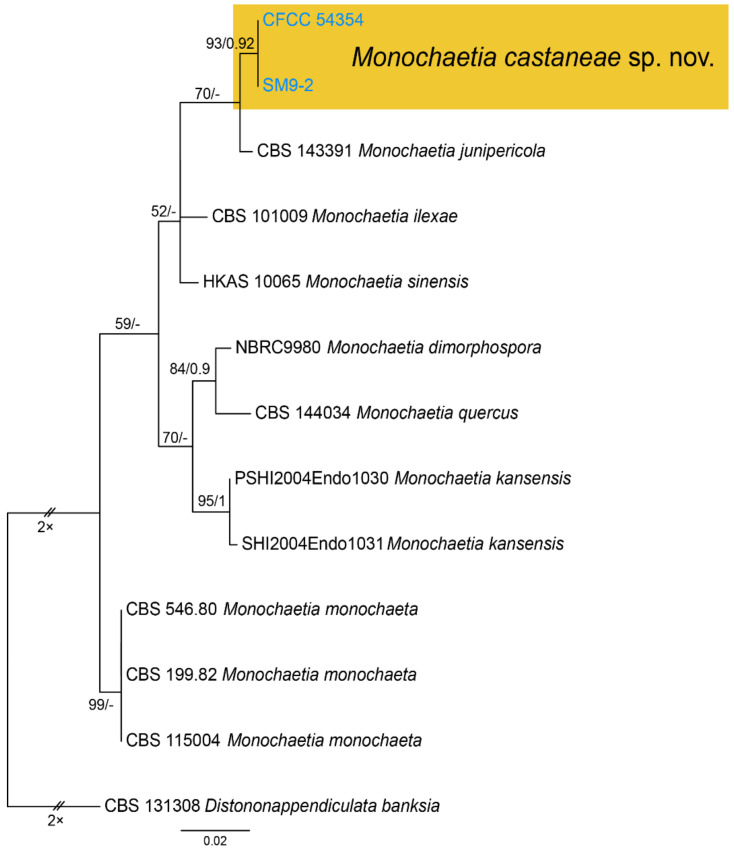
Phylogram generated from RAxML analysis based on combined ITS sequence data of *Monochaeta* isolates. The tree was rooted to *Distononappendiculata banksia* (CBS 131308). The scale bar indicates 0.02 nucleotide changes per site. Isolates from this study are highlighted. Isolates from this study are marked in blue, and the identified species is marked in yellow.

**Figure 12:**
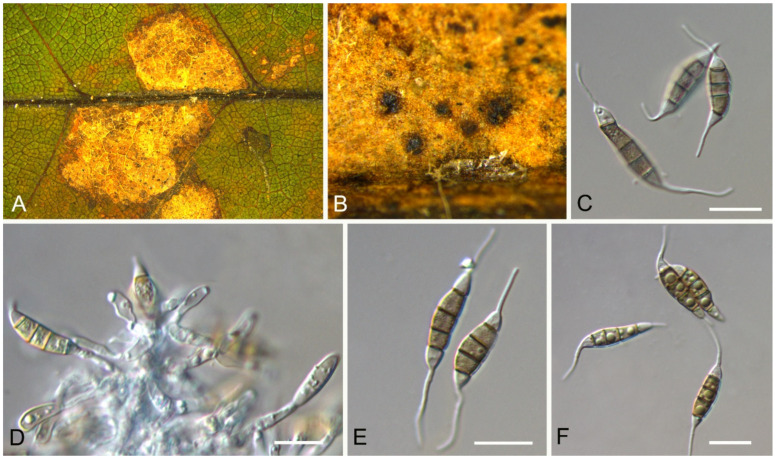
*Monochaetia castaneae* (BJFC-S1807). (**A**,**B**) Conidiomata on the diseased leaves of *Castanea mollissima*; (**C**,**E**,**F**) Conidia; (**D**) Conidiogenous cells giving rise to conidia. Scale bars: C–F = 10 μm.

Notes—*Monochaetia concentrica* and *M. kansensis* were recorded to inhabit *Castanea* leaves, but *Monochaetia castaneae* from present study is narrower than them (4.7–6.6 μm in *M. castaneae* vs. 6.5–8.5 μm in *M. concentrica* vs. 6.0–8.0 μm in *M. kansensis*) [47]. *Monochaetia castaneae* is phylogenetically close to *M. junipericola* from twigs of *Juniperus communis* (Figure 11), but they are distinguished by hosts and conidial sizes (18.8–27.3 × 4.7–6.6 μm in *M. castaneae* vs. 22–28 × 5.0–7.0 μm in *M. junipericola*) [48].

### 3.10. Neopestalotiopsis *Maharachch., K.D. Hyde & Crous*

*Neopestalotiopsis asiatica* Maharachch., K.D. Hyde & Crous, Stud. Mycol. 79: 136 (2014) (Figure 14)

Pathogenic on *Castanea mollissima* leaves. Sexual morph: Undetermined. Asexual morph: Conidiomata 80–200 μm diam., 20–150 μm high, acervular, conspicuous, scattered or aggregated, rounded, black. Conidiophores reduced to conidiogenous cells, smooth, hyaline. Conidiogenous cells 4–10 × 1.5–4 μm, discrete, thin-walled, lageniform, subcylindrical or irregular. Conidia (15.4–)17.6–23.1(–25.5) × (4.6–)5.4–7.8(–8.4) μm ($\bar{x}\;$= 19.4 × 6.2 μm, *n* = 50), L/W = (2.2–)2.7–3.5(–4) ($\bar{x}$ = 3.1, *n* = 50), basal cell conic to obconic with a truncate base, hyaline, minutely verruculose and thin-walled, 4–5.5 μm long; three median cells doliiform, versicolourous, second cell from base pale brown to olivaceous, 4.5–6 μm long; third cell honey brown, 5–6.5 μm long; fourth cell brown, 4.5–5.5 μm long; apical cell 3.5–5 μm long, hyaline, subcylindrical, rugose and thin-walled; with 3 (seldom 4) tubular apical appendages, arising from the apical crest, unbranched, filiform, 23–35 μm long; basal appendage present, 3.5–8.5 μm long.

Culture characteristics—Colonies on PDA reaching up to 60 mm in 10 days, dense aerial mycelium on the surface with undulate edge, white. Fruiting bodies were observed after 20 days.

Material examined—CHINA, Sichuan Province, Yaan City, Shimian County, 29°13′31″ N, 102°21′27″ E, alt. 978 m, on leaf spots of *Castanea mollissima*, 10 September 2020, N. Jiang (BJFC-S1789, living culture, CFCC 54339 = SM32); *ibid*. (living cultures, SM7, SM8B, SM11, SM19B).

**Figure 13:**
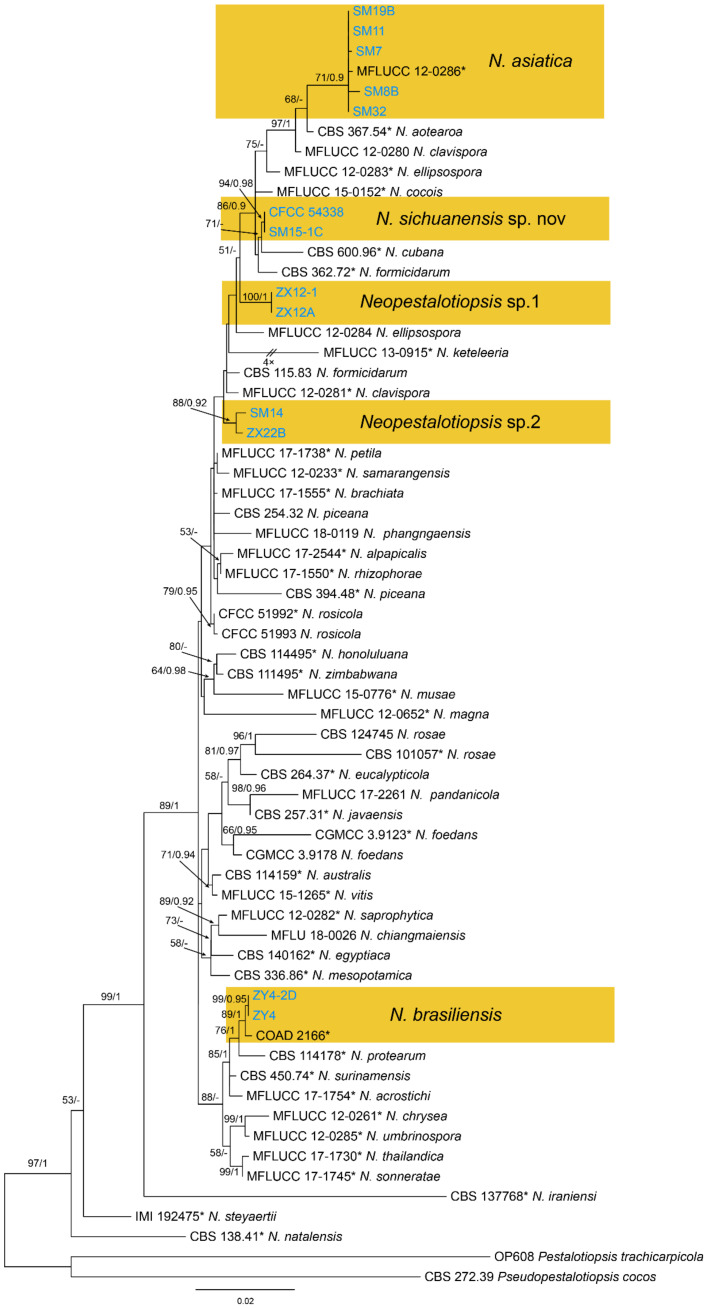
Phylogram generated from RAxML analysis based on combined ITS *tef1* and *tub2* sequence data of *Neopestalotiopsis* isolates. The tree was rooted to *Pestalotiopsis trachicarpicola* (OP608) and *Pseudopestalotiopsis cocos* (CBS 272.39). The scale bar indicates 0.02 nucleotide changes per site. Isolates from this study are marked in blue, ex-type strains are marked with *, and the identified species are marked in yellow.

**Figure 14:**
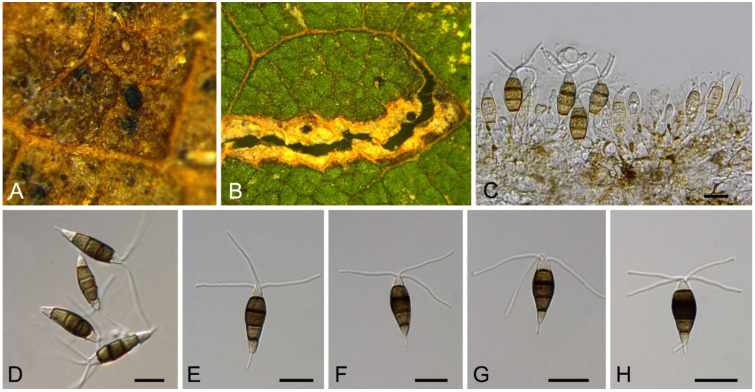
*Neopestalotiopsis asiatica* (BJFC-S1789). (**A**,**B**) Conidiomata on the diseased leaves of *Castanea mollissima*; (**C**) Conidiogenous cells giving rise to conidia; (**D**–**H**) Conidia. Scale bars: C–H = 10 μm.

Notes—*Neopestalotiopsis asiatica* was described based on the endophytic isolate from unknown trees in China under the name of *Pestalotiopsis asiatica* [49], but subsequently transferred to the genus *Neopestalotiopsis* [50]. In the present study, several isolates of *N. asiatica* were obtained from Chinese chestnut diseased leaves (Figure 13).

#### 3.10.1. *Neopestalotiopsis brasiliensis*

**Figure 15:**
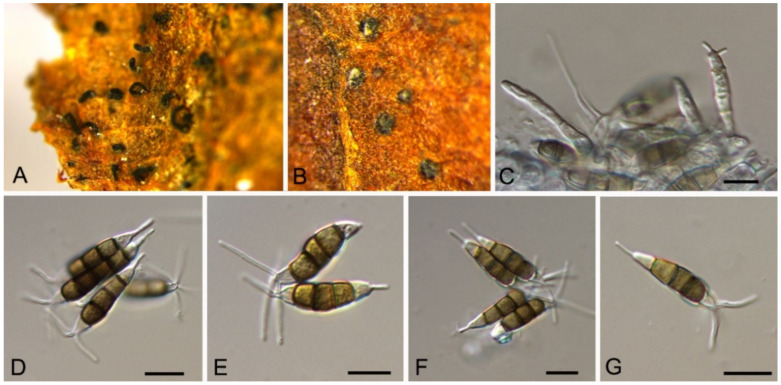
*Neopestalotiopsis brasiliensis* (BJFC-S1791). (**A**,**B**) Conidiomata on the diseased leaves of *Castanea mollissima*; (**C**) Conidiogenous cells giving rise to conidia; (**D**–**G**) Conidia. Scale bars: C–G = 10 μm.

*Neopestalotiopsis brasiliensis* V.P. Abreu, A.W.C. Rosado & O.L. Pereira, Acta Bot. Brasilica 32(4): 661 (2018) (Figure 15)

Pathogenic on *Castanea mollissima* leaves. Sexual morph: Undetermined. Asexual morph: Conidiomata 50–150 μm diam., 30–90 μm high, acervular, conspicuous, scattered or aggregated, rounded, black. Conidiophores reduced to conidiogenous cells, smooth, hyaline. Conidiogenous cells 6–15 × 1.5–3.5 μm, discrete, thin-walled, lageniform, subcylindrical or irregular. Conidia (18.5–)19.5–24.1(–26.1) × (5–)5.3–6.4(–6.8) μm ($\bar{x}\;$= 21.8 × 5.9 μm, *n* = 50), L/W = (2.9–)3.2–4.3(–4.4) ($\bar{x}\;$= 3.8, *n* = 50), fusoid, ellipsoid to subcylindrical, straight to slightly curved, 4-septate; basal cell conic to obconic with a truncate base, hyaline, minutely verruculose and thin-walled, 3–5.5 μm long; three median cells doliiform, versicolourous, second cell from base pale brown to olivaceous, 4–6.5 μm long; third cell honey brown, 4.5–5.5 μm long; fourth cell brown, 4.5–6 μm long; apical cell 3.5–5.5 μm long, hyaline, subcylindrical, rugose and thin-walled; with 2 (seldom 3) tubular apical appendages, arising from the apical crest, unbranched, filiform, 8–20 μm long; basal appendage present, 2–5.5 μm long.

Culture characteristics—Colonies on PDA reaching up to 60 mm in 7 days, dense aerial mycelium on the surface with undulate edge, white. Fruiting bodies were observed after 15 days.

Material examined—CHINA, Guizhou Province, Zunyi City, Goujiang Town, 27°24′49″ N, 106°52′49″ E, alt. 1064 m, on leaf spots of *Castanea mollissima*, 7 September 2020, N. Jiang (BJFC-S1791, living culture, CFCC 54341 = ZY4); *ibid*. (living culture, ZY4-2D).

Notes—*Neopestalotiopsis brasiliensis* was described from rotted fruits of *Psidium guajava* in Brazil [51]. In present study, strains from diseased chestnut leaves formed a supported clade with the ex-type strain COAD 2166 (Figure 13), and shared similar morphology. Hence, we identified our strains as *N. brasiliensis*, which represented a new host and geographical record.

#### 3.10.2. *Neopestalotiopsis sichuanensis*

**Figure 16:**
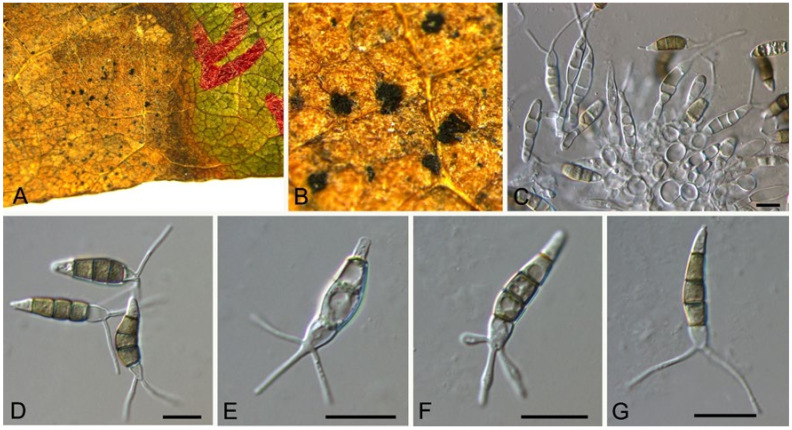
*Neopestalotiopsis sichuanensis* (BJFC-S1788). (**A**,**B**) Conidiomata on the diseased leaves of *Castanea mollissima*; (**C**) Conidiogenous cells giving rise to conidia; (**D**–**G**) Conidia. Scale bars: C–G = 10 μm.

C.M. Tian & N. Jiang, sp. nov. (Figure 16)

MycoBank: MB 837792

Etymology—named for the location of the type specimen.

Holotype—BJFC-S1788

Pathogenic on *Castanea mollissima* leaves. Sexual morph: Undetermined. Asexual morph: Conidiomata 100–250 μm diam., 50–150 μm high, acervular, conspicuous, scattered or aggregated, rounded, black. Conidiophores reduced to conidiogenous cells, smooth, hyaline. Conidiogenous cells 7–20 × 2–6 μm, discrete, thin-walled, lageniform, subcylindrical or irregular. Conidia (23.2–)24.3–30.4(–32.8) × (5.7–)6.3–7.1(–7.5) μm ($\bar{x}$ = 27.3 × 6.7 μm, *n* = 50), L/W = (3.4–)3.5–4.6(–5.1) ($\bar{x}$ = 4.1, *n* = 50), fusoid, ellipsoid to subcylindrical, straight to slightly curved, 4-septate; basal cell conic to obconic with a truncate base, hyaline, minutely verruculose and thin-walled, 3.5–5 μm long; three median cells doliiform, versicolourous, second cell from base pale brown to olivaceous, 3.5–6 μm long; third cell honey brown, 4.5–6.5 μm long; fourth cell brown, 4.5–6 μm long; apical cell 3.5–6 μm long, hyaline, subcylindrical, rugose and thin-walled; with 2 or 3 tubular apical appendages, arising from the apical crest, unbranched, filiform, 8–15 μm long; basal appendage present, 1.5–4 μm long.

Culture characteristics—Colonies on PDA reaching up to 60 mm in 7 days, dense aerial mycelium on the surface with undulate edge, white. Fruiting bodies were observed after 15 days.

Material examined—CHINA, Sichuan Province, Yaan City, Shimian County, 29°13′31″ N, 102°21′27″ E, alt. 978 m, on leaf spots of *Castanea mollissima*, 10 September 2020, N. Jiang (BJFC-S1788, holotype; ex-type living culture, CFCC 54338 = SM15-1); *ibid*. (living culture, SM15-1C).

Notes—*Neopestalotiopsis sichuanensis* was phylogenetically close to *N. cubana* (Figure 13) but differed in conidial width (5.7–7.5 μm in *N. sichuanensis* vs. 7.5–10 μm in *N. cubana*) [50].

#### 3.10.3. *Neopestalotiopsis* sp.1

**Figure 17:**
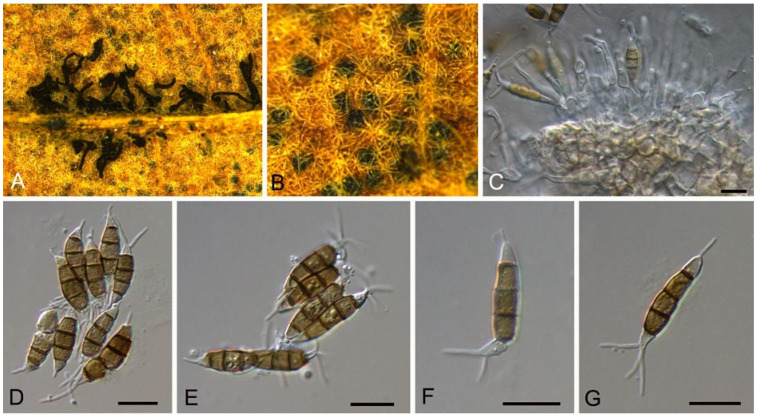
*Neopestalotiopsis* sp.1 (BJFC-S1787). (**A**,**B**) Conidiomata on the diseased leaves of *Castanea mollissima*; (**C**) Conidiogenous cells giving rise to conidia; (**D**–**G**) Conidia. Scale bars: C–G = 10 μm.

Pathogenic on *Castanea mollissima* leaves. Sexual morph: Undetermined. Asexual morph: Conidiomata 80–150 μm diam., 30–60 μm high, acervular, conspicuous, scattered or aggregated, rounded, black. Conidiophores reduced to conidiogenous cells, smooth, hyaline. Conidiogenous cells 5.5–15.5 × 3–7 μm, discrete, thin-walled, lageniform, subcylindrical or irregular. Conidia (19.1–)19.9–23.2(–24.7) × (5.4–)5.8–7.6(–8.6) μm ($\bar{x}\;$= 21.6 × 6.7 μm, *n* = 50), L/W = (2.9–)3.0–3.5(–3.7) ($\bar{x}$ = 3.2, *n* = 50), fusoid, ellipsoid to subcylindrical, straight to slightly curved, 4-septate; basal cell conic to obconic with a truncate base, hyaline, minutely verruculose and thin-walled, 3–4.5 μm long; three median cells doliiform, versicolourous, second cell from base pale brown to olivaceous, 3–5 μm long; third cell honey brown, 4.5–6 μm long; fourth cell brown, 4–6 μm long; apical cell 3.5–5 μm long, hyaline, subcylindrical, rugose and thin-walled; with 2 or 3 tubular apical appendages, arising from the apical crest, unbranched, filiform, 7.5–14 μm long; basal appendage present, 3–6.5 μm long (Figure 17).

Culture characteristics—Colonies on PDA reaching up to 60 mm in 7 days, dense aerial mycelium on the surface with undulate edge, white. Fruiting bodies were observed after 15 days.

Material examined—CHINA, Yunnan Province, Zhaotong City, Zhenxiong County, 27°43′28″ N, 105°10′35″ E, alt. 1280 m, on leaf spots of *Castanea mollissima*, 5 September 2020, N. Jiang (BJFC-S1787, living culture, CFCC 54337 = ZX12A); *ibid*. (living culture, ZX12-1).

Notes—Although phylogenetically distinct, these two isolates were not proposed as a new species for lack of distinguished characters from close clades (Figure 13).

#### 3.10.4. *Neopestalotiopsis* sp.2

**Figure 18:**
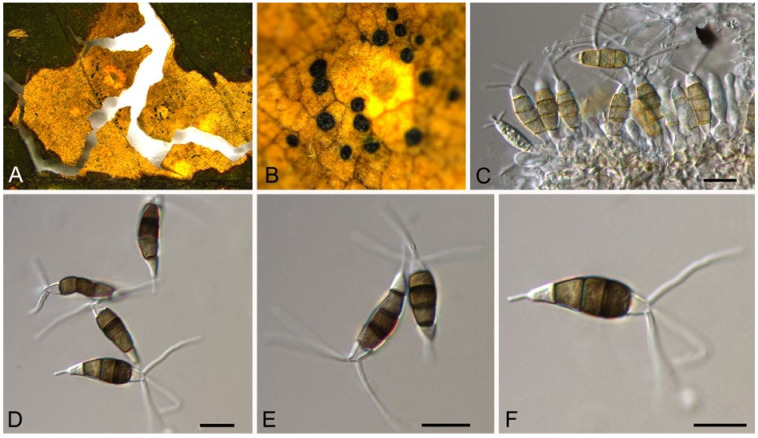
*Neopestalotiopsis* sp.2 (BJFC-S1790). (**A**,**B**) Conidiomata on the diseased leaves of *Castanea mollissima*; (**C**) Conidiogenous cells giving rise to conidia; (**D**–**F**) Conidia. Scale bars: C–F = 10 μm.

Pathogenic on *Castanea mollissima* leaves. Sexual morph: Undetermined. Asexual morph: Conidiomata 75–175 μm diam., 30–75 μm high, acervular, conspicuous, scattered or aggregated, rounded, black. Conidiophores reduced to conidiogenous cells, smooth, hyaline. Conidiogenous cells 3.5–8 × 2.5–5.5 μm, discrete, thin-walled, lageniform, subcylindrical or irregular. Conidia (21.4–)22–25.2(–26.2) × (5.1–)6.2–7.7(–8.7) μm ($\bar{x}\;$= 23.6 × 7 μm, *n* = 50), L/W = (2.9–)3–3.9(–4.4) ($\bar{x}\;$= 3.4, *n* = 50), fusoid, ellipsoid to subcylindrical, straight to slightly curved, 4-septate; basal cell conic to obconic with a truncate base, hyaline, minutely verruculose and thin-walled, 3–5 μm long; three median cells doliiform, versicolourous, second cell from base pale brown to olivaceous, 4.5–6.5 μm long; third cell honey brown, 4.5–6 μm long; fourth cell brown, 4–6 μm long; apical cell 3.5–5 μm long, hyaline, subcylindrical, rugose and thin-walled; with 2 (seldom 3) tubular apical appendages, arising from the apical crest, unbranched, filiform, 10–25 μm long; basal appendage present, 1.5–5 μm long (Figure 18).

Culture characteristics—Colonies on PDA reaching up to 60 mm in 7 days, dense aerial mycelium on the surface with undulate edge, white. Fruiting bodies were observed after 15 days.

Material examined—CHINA, Sichuan Province, Yaan City, Shimian County, 29°13′31″ N, 102°21′27″ E, alt. 978 m, on leaf spots of *Castanea mollissima*, 10 September 2020, N. Jiang (BJFC-S1790, living culture, CFCC 54340 = SM14); Yunnan Province, Zhaotong City, Zhenxiong County, 27°43′28″ N, 105°10′35″ E, alt. 1280 m, on leaf spots of *Castanea mollissima*, 5 September 2020, N. Jiang (living culture, ZX22B).

Notes—Although phylogenetically distinct, these two isolates were not proposed as a new species for lack of distinguished characters from close clades (Figure 13).

### 3.11. Pestalotiopsis *Steyaert*

**Figure 19:**
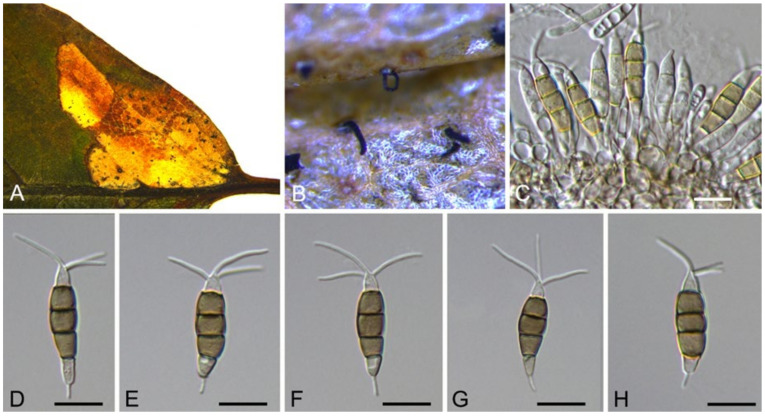
*Pestalotiopsis kenyana* (BJFC-S1784). (**A**,**B**) Conidiomata on the diseased leaves of *Castanea mollissima*; (**C**) Conidiogenous cells giving rise to conidia; (**D**–**H**) Conidia. Scale bars: C–H = 10 μm.

*Pestalotiopsis kenyana* Maharachch., K.D. Hyde & Crous, Stud. Mycol. 79: 166 (2014) (Figure 19)

Pathogenic on *Castanea henryi* and *C. mollissima* leaves. Sexual morph: Undetermined. Asexual morph: Conidiomata 50–250 μm diam., 30–150 μm high, acervular, conspicuous, scattered or aggregated, rounded, black. Conidiophores reduced to conidiogenous cells, smooth, hyaline. Conidiogenous cells 5–19 × 2.5–6.5 μm, discrete, thin-walled, lageniform, subcylindrical or irregular. Conidia (20.4–)21.8–26.2(–28) × (6.1–)6.3–7.4(–8) μm ($\bar{x}\;$= 23.8 × 6.9 μm, *n* = 50), L/W = (3–)3.1–3.9(–4.2) ($\bar{x}\;$= 3.5, *n* = 50), fusoid, ellipsoid to subcylindrical, straight to slightly curved, 4-septate; basal cell conic to obconic with a truncate base, hyaline, minutely verruculose and thin-walled, 3–5 μm long; three median cells doliiform, concolourous, brown, second cell 3–5.5 μm long; third cell 4.5–6.5 μm long; fourth cell 4.5–6 μm long; apical cell 3.5–5.5 μm long, hyaline, subcylindrical, rugose and thin-walled; with 3 tubular apical appendages, arising from the apical crest, unbranched, filiform, 3.5–15 μm long; basal appendage present, 1.5–3.5 μm long.

Culture characteristics—Colonies on PDA reaching up to 60 mm in 7 days, dense aerial mycelium on the surface with undulate edge, white. Fruiting bodies were observed after 15 days.

Material examined—CHINA, Yunnan Province, Zhaotong City, Zhenxiong County, 27°43′28″ N, 105°10′35″ E, alt. 1280 m, on leaf spots of *Castanea mollissima*, 5 September 2020, N. Jiang (BJFC-S1784, living culture, CFCC 54336 = ZX11); *ibid*. (living culture, ZX3, ZX7, ZX9, ZX18A); Guizhou Province, Zunyi City, Goujiang Town, 27°24′49″ N, 106°52′49″ E, alt. 1064 m, on leaf spots of *Castanea mollissima*, 7 September 2020, N. Jiang (BJFC-S1786, living culture, ZY6-2A); *ibid*. (living culture, ZY7); Sichuan Province, Yaan City, Shimian County, 29°13′31″ N, 102°21′27″ E, alt. 978 m, on leaf spots of *Castanea mollissima*, 10 September 2020, N. Jiang (BJFC-S1785, living culture, SM18); Hunan Province, Changsha City, Changsha County, Jinjing Town, 28°58′52″ N, 113°34′38″ E, alt. 63 m, on leaf spots of *Castanea henryi*, 10 November 2020, C.M. Tian & N. Jiang (BJFC-S1817; living culture, JJ 2A); *ibid*. (living cultures, JJ5, JJ10, JJ13, JJ15, JJ17, JJ18, JJ26).

**Figure A7:**
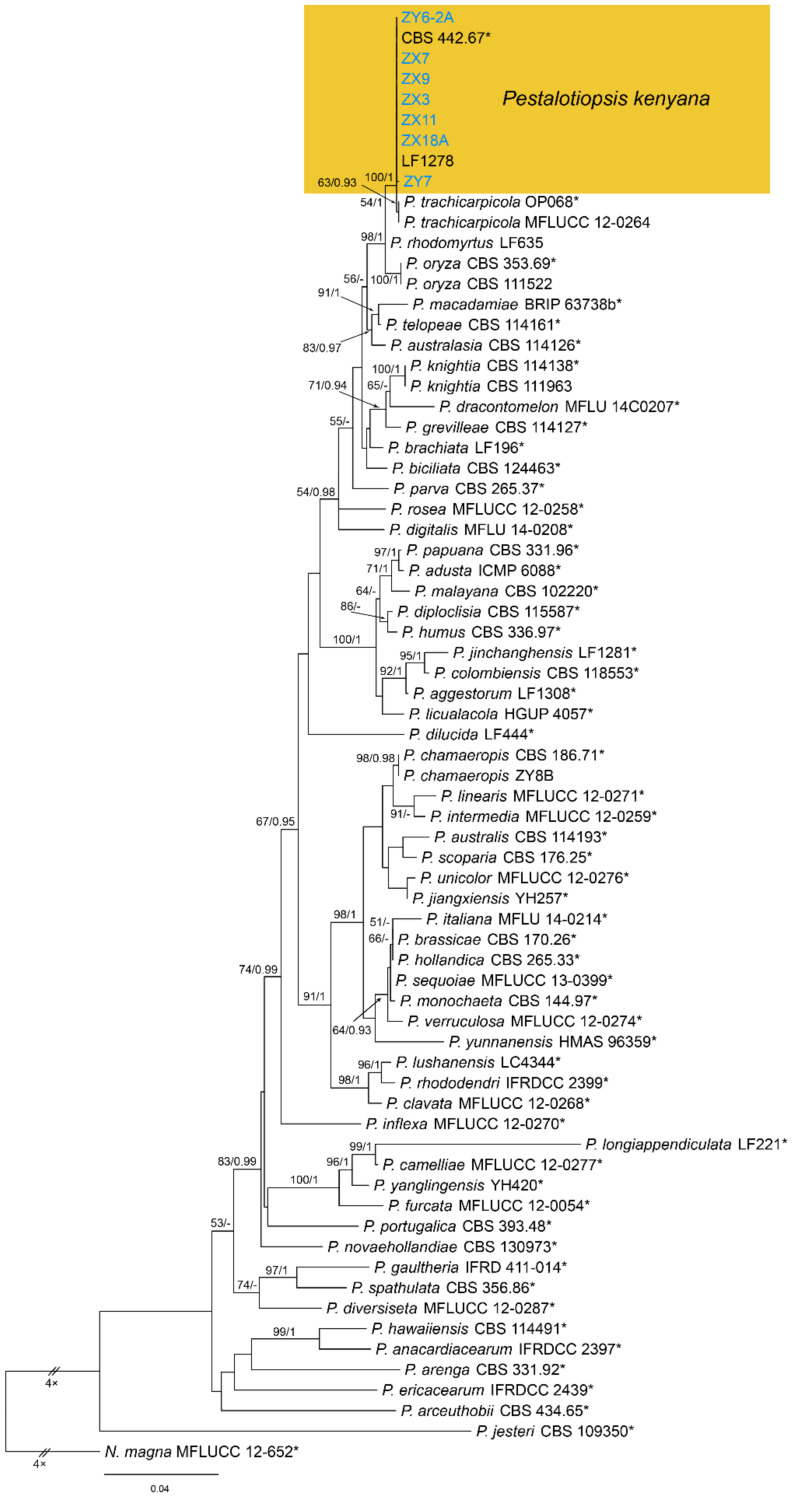
Phylogram generated from RAxML analysis based on combined ITS, *tef1* and *tub2* sequence data of *Pestalotiopsis* isolates. The tree was rooted to *Neopestalotiopsis magna* (MFLUCC 12-652). The scale bar indicates 0.04 nucleotide changes per site. Isolates from this study are marked in blue, ex-type strains are marked with *, and the identified species is marked in yellow.

Notes—*Pestalotiopsis kenyana* was proposed from *Coffea* sp. in Kenya [50]. Strains collected from *Castanea mollissima* in present study formed a supported clade with *Pestalotiopsis kenyana* (Figure A7), and shared similar morphology. Hence, we identified our strains as *P. kenyana*, which represented a new host record.

### 3.12. Robillarda *Sacc.*

**Figure 20:**
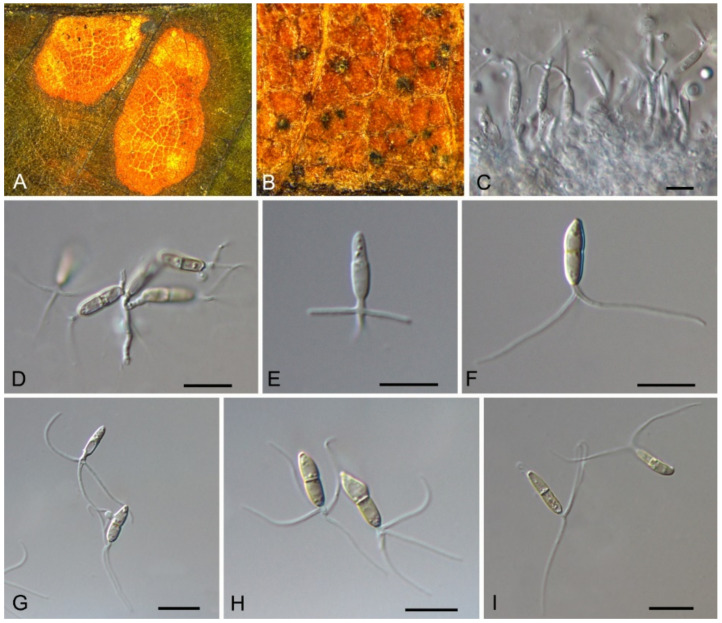
*Robillarda sessilis* (BJFC-S1804). (**A**,**B**) Conidiomata on the diseased leaves of *Castanea mollissima*; (**C**,**D**) Conidiogenous cells giving rise to conidia; (**E**–**I**) Conidia. Scale bars: C–I = 10 μm.

*Robillarda sessilis* (Sacc.) Sacc., Michelia 2(no. 6): 8 (1880) (Figure 20)

Pathogenic on *Castanea mollissima* leaves. Sexual morph: Undetermined. Asexual morph: Conidiomata 100–200 μm diam., 20–60 μm high, acervular, conspicuous, scattered or aggregated, rounded, black. Conidiophores reduced to conidiogenous cells, smooth, hyaline. Conidiogenous cells 2.5–6 × 1.5–3 μm, discrete, thin-walled, guttulate or not, lageniform, ampulliform or irregular. Conidia composed of a 1-septate conidium body and a septate apical cell modified into a branched appendage. Conidium body (10.7–)11.2–13.1(–13.5) × (2.9–)3.1–3.8(–3.9) μm ($\bar{x}$ = 12.2 × 3.4 μm, *n* = 50), L/W = (2.9–)3.1–4.1(–4.5) ($\bar{x}$ = 3.6, *n* = 50), cylindrical, straight, 1-septate, smooth, hyaline to pale brown, guttulate, slightly constricted at the median septum; apical cell cylindrical for 4.0–5.0 μm long, then dividing into 2–4 (mostly 3) divergent branches; apical appendages unbranched, attenuated, 15–28 μm long; basal appendages absent.

Culture characteristics—Colonies on PDA reaching up to 60 mm in 15 days, flat with entire edge, white, aerial mycelia villiform. Fruiting bodies were observed after 20 days.

Material examined—CHINA, Yunnan Province, Zhaotong City, Zhenxiong County, 27°43′28″ N, 105°10′35″ E, alt. 1280 m, on leaf spots of *Castanea mollissima*, 5 September 2020, N. Jiang (BJFC-S1804, living culture, CFCC 54353 = ZX5); *ibid*. (living culture, ZX5-1); Guizhou Province, Zunyi City, Goujiang Town, 27°24′49″ N, 106°52′49″ E, alt. 1064 m, on leaf spots of *Castanea mollissima*, 7 September 2020, N. Jiang (living culture, ZY5-1).

**Figure A8:**
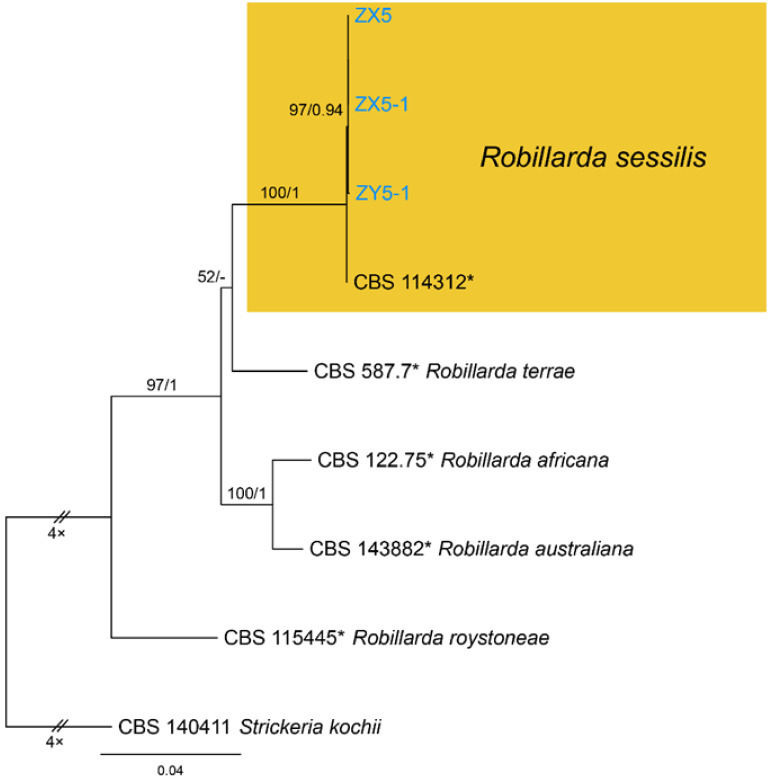
Phylogram generated from RAxML analysis based on combined ITS, LSU, *rpb2*, *tef1* and *tub2* sequence data of *Robillarda* isolates. The tree was rooted to *Strickeria kochii* (CBS 140411). The scale bar indicates 0.04 nucleotide changes per site. Isolates from this study are marked in blue, ex-type strains are marked with *, and the identified species is marked in yellow.

Notes—*Robillarda sessilis* was documented from quite variable hosts, such as *Bischofia*, *Cocos*, *Ficus*, *Fragaria*, *Fumana*, *Ludwigia*, *Magnolia*, *Paeonia*, *Quercus*, *Randia*, *Rosa*, *Rubus*, and *Vitis* [52]. Strains from present study clustered with the ex-epitype strain (CBS 114312) of *Robillarda sessilis* (Figure A8). This is the first report of this fungus on the host *Castanea mollissima*, and in China as a country.

### 3.13. Diaporthe *Nitschke*

**Figure 21:**
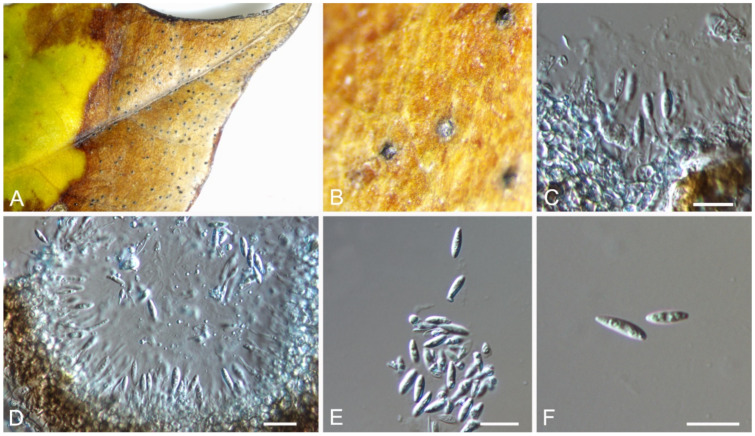
*Diaporthe lithocarpi* (BJFC-S1809). (**A**,**B**) Conidiomata on the diseased leaves of *Castanea henryi*; (**C**) Conidiogenous cells giving rise to conidia; (**D**) Section through the pycnidium; (**E**,**F**) Conidia. Scale bars: C–F = 10 μm.

*Diaporthe lithocarpi* Y.H. Gao & L. Cai, Fungal Biology 119(5): 306 (2015) (Figure 21)

Pathogenic on *Castanea henryi* leaves. Sexual morph: Undetermined. Asexual morph: Conidiomata 120–3000 μm diam., pycnidial, aggregated, globose to subglobose, black. Conidiophores reduced to conidiogenous cells. Conidiogenous cells 2.5–12.5 × 1.5–3 μm, cylindrical, hyaline, phiailidic, unbranched, straight. Conidia (7.6–)7.9–9.1(–9.8) × (2.5–)2.6–3(–3.1) μm ($\bar{x}\;$= 8.5 × 2.8 μm, *n* = 50), L/W = (2.4–)2.7–3.4(–3.7) ($\bar{x}\;$= 3.1, *n* = 50), aseptate, hyaline, ellipsoidal, biguttulate, mostly with one end obtuse and the other acute.

Culture characteristics—Colonies on PDA reaching up to 60 mm in 7 days, flat, initially white mycelium, gradually becoming pale brownish, with cottony aerial mycelium and fringed margin; reverse pale yellowish.

Material examined—CHINA, Hunan Province, Changsha City, Changsha County, Jinjing Town, 28°58′52″ N, 113°34′38″ E, alt. 63 m, on leaf spots of *Castanea henryi*, 10 November 2020, C.M. Tian & N. Jiang (BJFC-S1809; living culture, CFCC 54573 = JJ3); *ibid*. (living cultures, JJ3-2, JJ26B).

**Figure A9:**
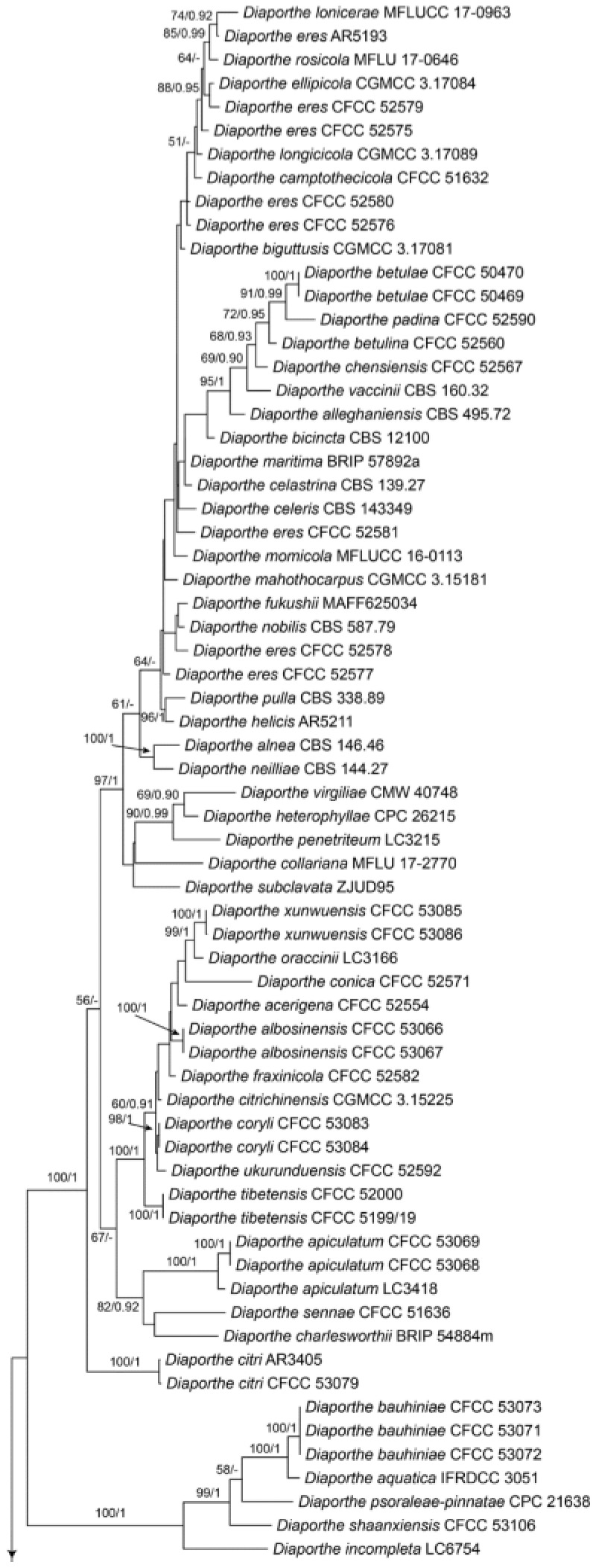

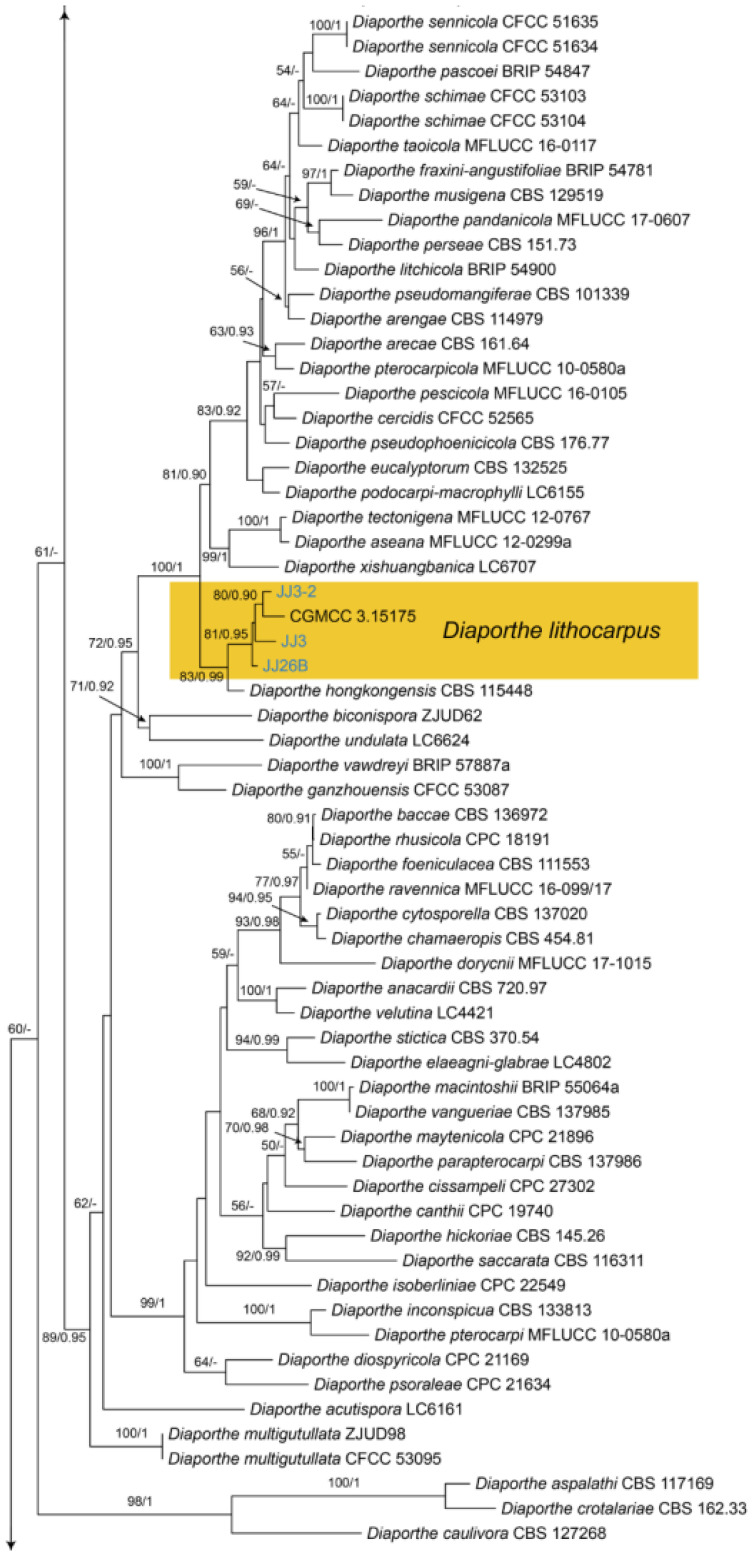

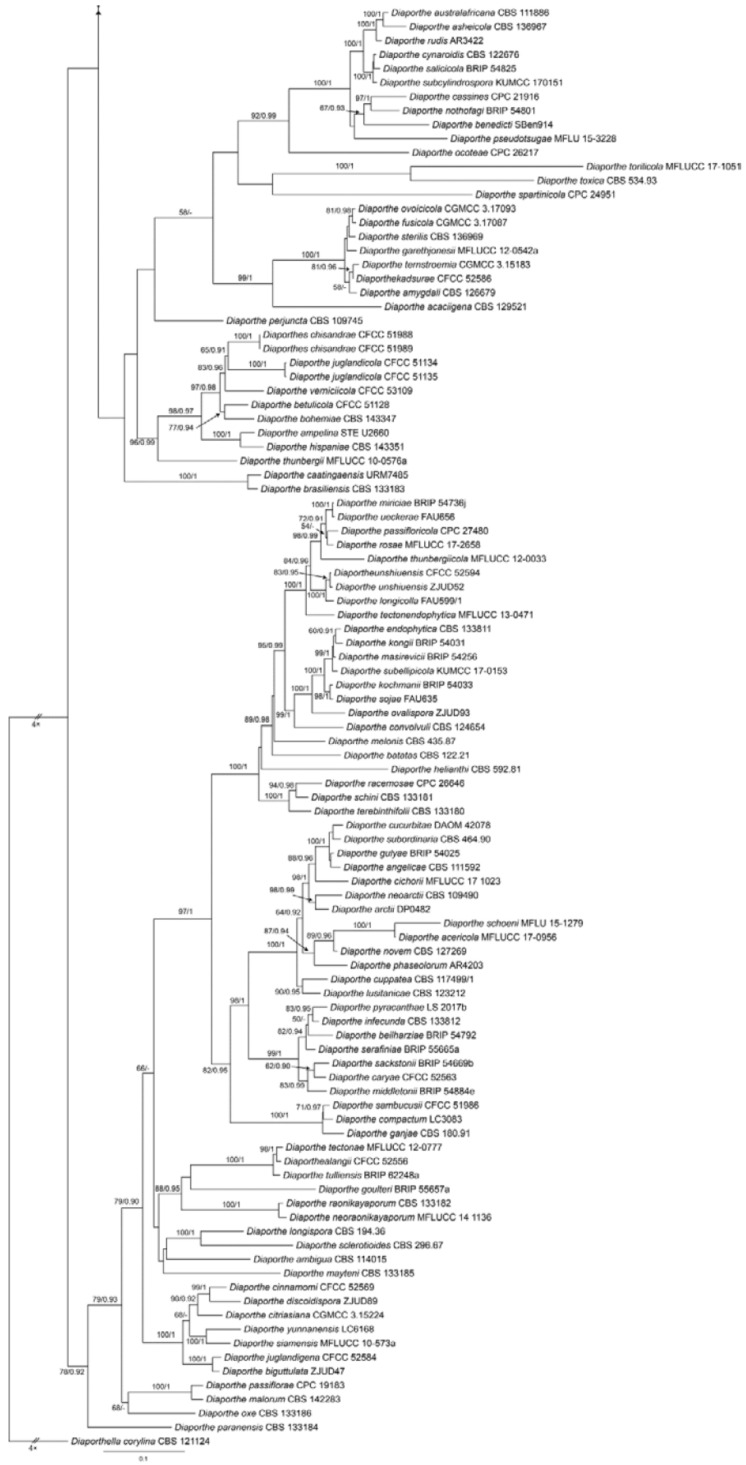
Phylogram generated from RAxML analysis based on combined ITS, *cal*, *his*, *tef1* and *tub2* sequence data of *Diaporthe* isolates. The tree was rooted to *Diaporthella corylina* (CBS 121124). The scale bar indicates 0.1 nucleotide changes per site. Isolates from this study are marked in blue, and the identified species is marked in yellow.

Notes—*Diaporthe lithocarpi* was reported to cause leaf spots on *Lithocarpus glabra*, *Loropetalum chinensis*, *Smilax china*, *S. glabra* and *Ternstroemia gymnanthera* in Zhejiang Province of China [53]. The present study adds a new *Castanea henryi* host for the fungus based on the phylogenetic inference of ITS, *cal*, *his*, *tef1* and *tub2* sequence data (Figure A9), and exactly matched morphology.

### 3.14. Gnomoniopsis *Berl.*

**Figure 22:**
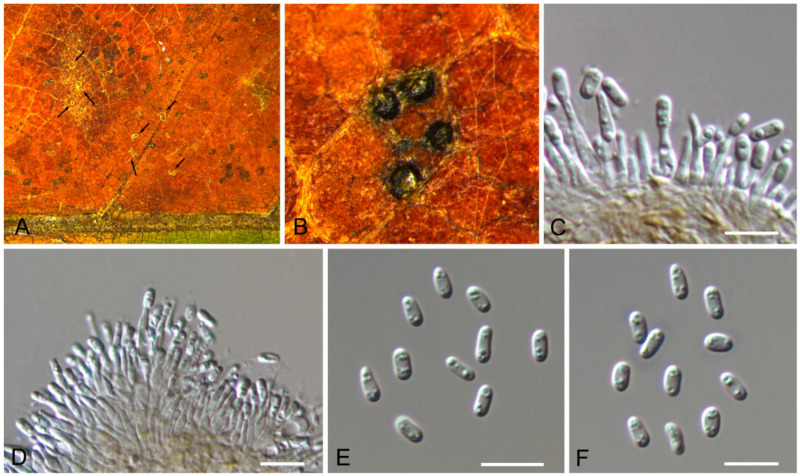
*Gnomoniopsis daii* (BJFC-S1794). (**A**,**B**) Conidiomata on the diseased leaves of *Castanea mollissima* (arrows showing orange tendrils); (**C**,**D**) Conidiogenous cells giving rise to conidia; (**E**,**F**) Conidia. Scale bars: C–F = 10 μm.

*Gnomoniopsis daii* C.M. Tian & N. Jiang, Forests 10(11/1016): 6 (2019) (Figure 22)

Pathogenic on *Castanea mollissima* leaves. Sexual morph: Undetermined. Asexual morph: Conidiomata 30–100 μm diam., pycnidial, aggregated, globose to pulvinate, black, single ostiolate, forming long and yellow tendrils. Conidiophores reduced to conidiogenous cells. Conidiogenous cells 2.5–18 × 1.5–3 μm, cylindrical, hyaline, phiailidic, unbranched, straight. Conidia (4.9–)5.1–5.9(–6.2) × (2.4–)2.5–2.6(–2.7) μm ($\bar{x}\;$= 5.5 × 2.5 μm, *n* = 50), L/W = (1.9–)2–2.2(–2.5) ($\bar{x}\;$= 2.2, *n* = 50), aseptate, hyaline, ellipsoidal, guttulate.

Culture characteristics—Colonies on PDA reaching up to 60 mm in 7 days, flat, velutinous to shortly woolly, dark brown in center, gradually lightening to pale grey at margin; margin diffuse; reverse dark brown to grey.

Material examined—CHINA, Yunnan Province, Zhaotong City, Zhenxiong County, 27°43′28″ N, 105°10′35″ E, alt. 1280 m, on leaf spots of *Castanea mollissima*, 5 September 2020, N. Jiang (BJFC-S1794, living culture, ZX14-1); Guizhou Province, Zunyi City, Goujiang Town, 27°24′49″ N, 106°52′49″ E, alt. 1064 m, on leaf spots of *Castanea mollissima*, 7 September 2020, N. Jiang (BJFC-S1795, living culture, CFCC 54345 = ZY11); *ibid*. (living cultures, ZY10-1, ZY10-3, ZY12A).

**Figure A10:**
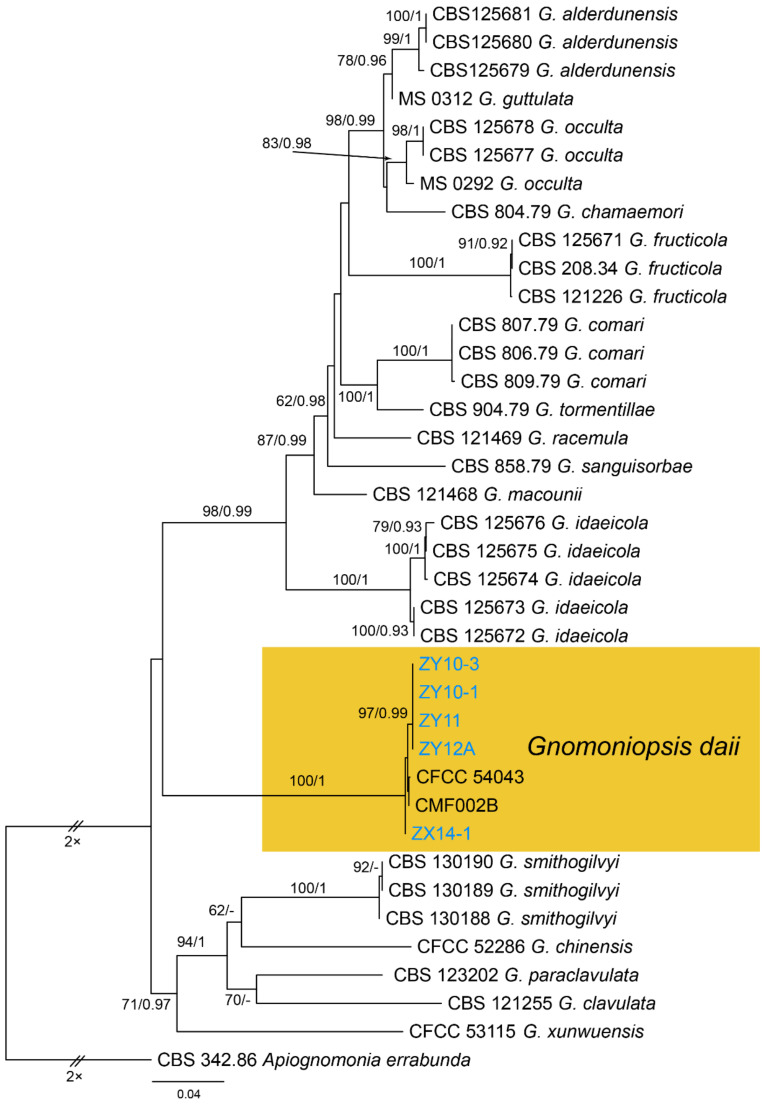
Phylogram generated from RAxML analysis based on combined ITS, *tef1* and *tub2* sequence data of *Gnomoniopsis* isolates. The tree was rooted to *Apiognomonia errabunda* (CBS 342.86). The scale bar indicates 0.04 nucleotide changes per site. Isolates from this study are marked in blue, and the identified species is marked in yellow.

Notes—The fungus *Gnomoniopsis smithogilvyi* causes fruit rot and branch canker diseases on *Castanea sativa* in Australia and Europe [4,5]. Interestingly, similar symptoms on *Castanea mollissima* in China were caused by two different species. *Gnomoniopsis chinese* causes branch canker, and *G. daii* causes fruit rot [54,55]. In this study, we confirmed the *Gnomoniopsis* pathogen on leaves in China as *G. daii* based on the phylogenetic inference of ITS, *tef1* and *tub2* sequence data (Figure A10), and exactly matched morphology.

### 3.15. *Pyrisporaceae C.M. Tian & N. Jiang*

#### 3.15.1. Pyrisporaceae C.M. Tian & N. Jiang, fam. nov.

MycoBank: MB 838315

Etymology—named from the type genus, *Pyrispora*.

Type genus—*Pyrispora* C.M. Tian & N. Jiang

Pathogenic or saprobic on *Castanea mollissima* leaves. Sexual morph: Ascomata semi-immersed, aggregated, globose to pulvinate, black, single ostiolate. Ostioles single, dark grey to black. Paraphyses deliquescent. Asci cylindrical to clavate, 8-spored, bi-seriate, with a distinct apical ring. Ascospores aseptate, hyaline, smooth, fusoid, multiguttulate, straight to slight curved. Asexual morph: Conidiomata pycnidial, aggregated, globose to subglobose, black, single ostiolate. Conidiophores reduced to conidiogenous cells. Conidiogenous cells pyriform base with long neck, hyaline, phiailidic, unbranched, straight. Conidia aseptate, hyaline, smooth, ellipsoidal, multiguttulate.

**Figure 23:**
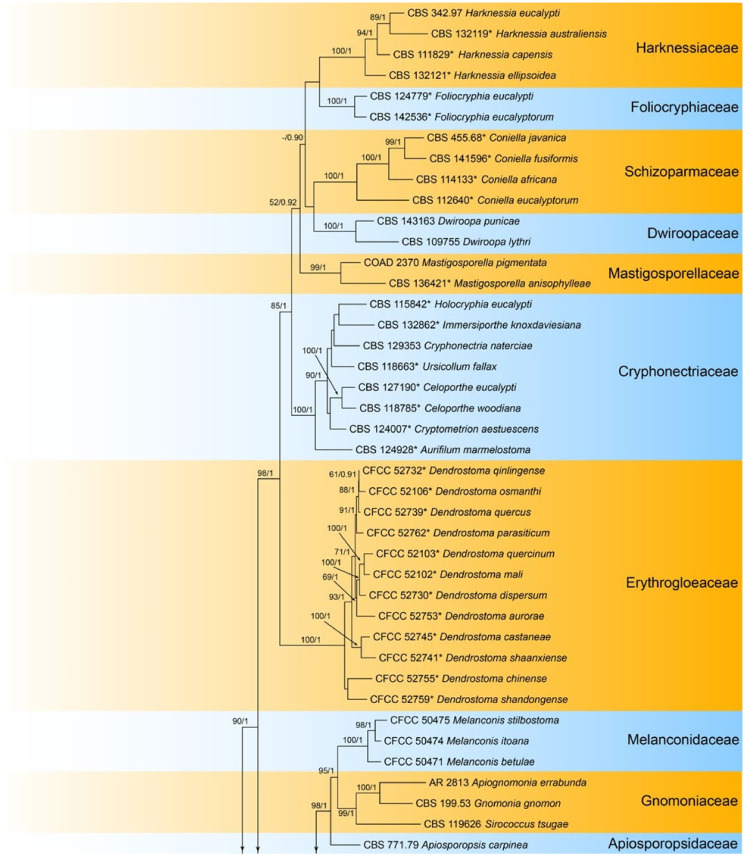

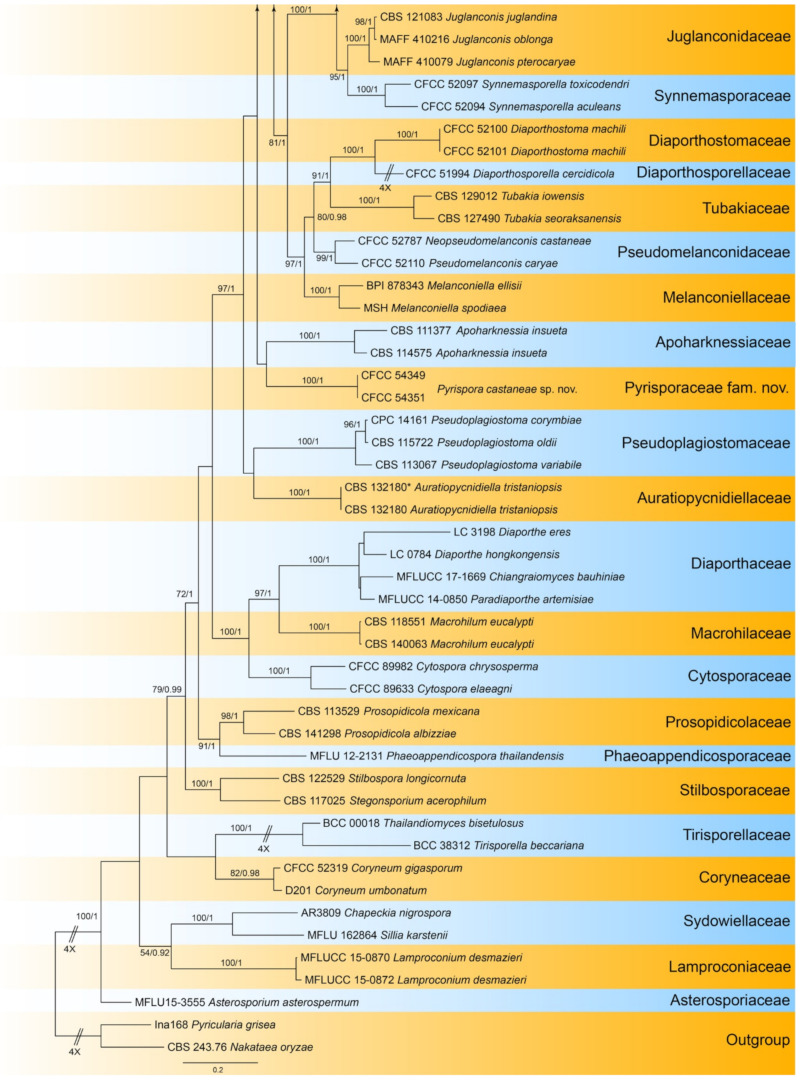
Phylogram generated from RAxML analysis based on combined ITS, LSU, *tef1* and *rpb2* sequence data of Diaporthales isolates. The tree was rooted to *Pyricularia grisea* (Ina 168) and *Nakataea oryzae* (CBS 243.76). The scale bar indicates 0.2 nucleotide changes per site.

Notes—The fungal order Diaporthales was well-classified based on both morphology and phylogeny in recent years (Figure 23) [56,57,58,59]. In this study, the sexual morph was observed on *Castanea* leaves, showing the typical characters of Diaporthales, the asci with distinct apical ring. Additionally, the asexual morph is distinctive based on the conidiogenous cells with pyriform base and long neck. Hence, we proposed a new family to accommodate this species.

#### 3.15.2. *Pyrispora* C.M. Tian & N. Jiang, gen. nov.

MycoBank: MB 838316

Etymology—named for the pyriform base of the conidiogenous cells.

Type species—*Pyrispora castaneae* C.M. Tian & N. Jiang

Pathogenic or saprobic on *Castanea mollissima* leaves. Sexual morph: Ascomata semi-immersed, aggregated, globose to pulvinate, black, single ostiolate. Ostioles single, dark grey to black. Paraphyses deliquescent. Asci cylindrical to clavate, 8-spored, bi-seriate, with a distinct apical ring. Ascospores aseptate, hyaline, smooth, fusoid, multiguttulate, straight to slight curved. Asexual morph: Conidiomata pycnidial, aggregated, globose to subglobose, black, single ostiolate. Conidiophores reduced to conidiogenous cells. Conidiogenous cells pyriform base with long neck, hyaline, phiailidic, unbranched, straight. Conidia aseptate, hyaline, smooth, ellipsoidal, multiguttulate.

#### 3.15.3. *Pyrispora castaneae* C.M. Tian & N. Jiang, sp. nov.

MycoBank: MB 838317

Etymology—named for the host genus, *Castanea*.

Holotype—BJFC-S1798

**Figure 24:**
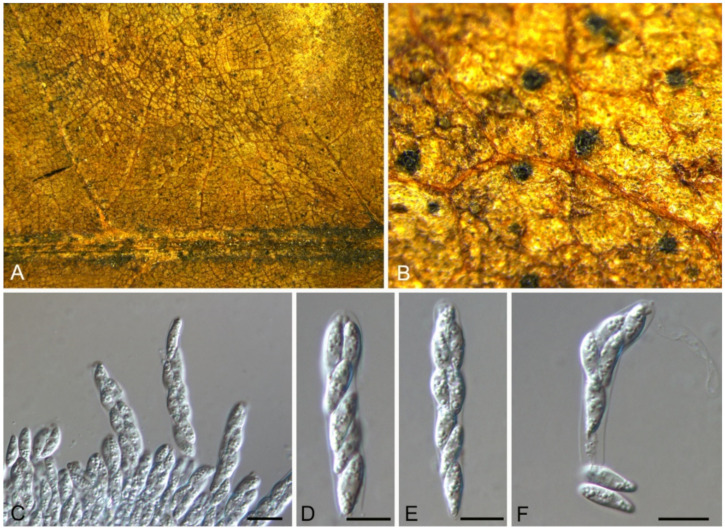
*Pyrispora castaneae* (BJFC-S1800). (**A**,**B**) Ascomata on the diseased leaves of *Castanea mollissima*; (**C**–**F**) Asci and ascospores. Scale bars: C–F = 10 μm.

**Figure 25:**
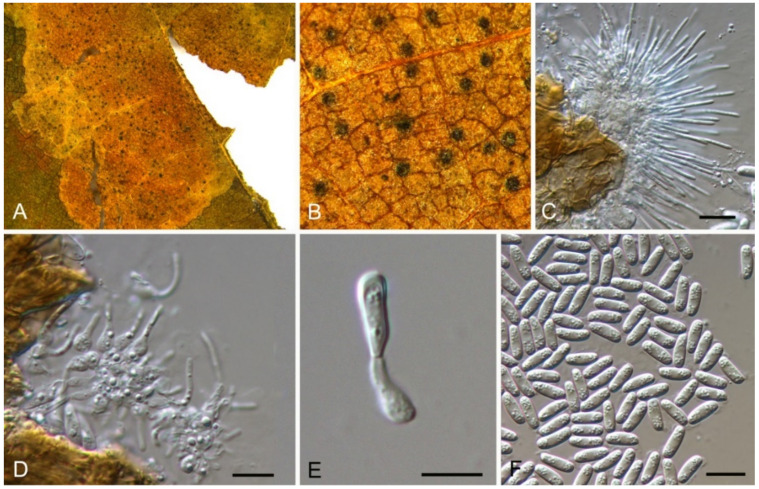
*Pyrispora castaneae* (BJFC-S1798). (**A**,**B**) Conidiomata on the diseased leaves of *Castanea mollissima*; (**C**–**E**) Conidiogenous cells giving rise to conidia; (**F**) Conidia. Scale bars: C–F = 10 μm.

Pathogenic or saprobic on *Castanea mollissima* leaves. Sexual morph: Ascomata 80–200 μm diam., semi-immersed, aggregated, globose to pulvinate, black, single ostiolate. Ostioles 30–75 μm, diam., single, dark grey to black. Paraphyses deliquescent. Asci (41–)44.5–52(–58) × (7–)8.5–10.5(–11) μm, cylindrical to clavate, 8-spored, bi-seriate, with a distinct apical ring. Ascospores (11.4–)12.2–14.5(–14.9) × (4.3–)4.4–4.9(–5.2) μm ($\bar{x}\;$= 13.3 × 4.7 μm, *n* = 50), L/W = (2.2–)2.5–3(–3.2) ($\bar{x}\;$= 2.9, *n* = 50), aseptate, hyaline, smooth, fusoid, multiguttulate, straight to slight curved. Asexual morph: Conidiomata 60–250 μm diam., pycnidial, aggregated, globose to subglobose, black, single ostiolate. Conidiophores reduced to conidiogenous cells. Conidiogenous cells 4–7.5 × 2–3.5 μm, pyriform base with long neck, necks up to 45 μm, hyaline, phiailidic, unbranched, straight. Conidia (10.4–)11.7–13(–13.9) × (4.1–)4.4–4.9(–5.5) μm ($\bar{x}\;$= 12.3 × 4.5 μm, *n* = 50), L/W = (2.2–)2.5–3.2(–3.4) ($\bar{x}$ = 2.8, *n* = 50), aseptate, hyaline, smooth, ellipsoidal, multiguttulate (Figure 24 and Figure 25).

Culture characteristics—Colonies on PDA reaching up to 60 mm in 7 days, flat, white, with cottony aerial mycelium and fringed margin; reverse pale yellowish.

Material examined—CHINA, Sichuan Province, Yaan City, Shimian County, 29°13′31″ N, 102°21′27″ E, alt. 978 m, on leaf spots of *Castanea mollissima*, 10 September 2020, N. Jiang (BJFC-S1798, holotype; ex-type living culture, CFCC 54349 = SM17); *ibid*. (BJFC-S1799, living culture, CFCC 54350 = SM20); *ibid*. (BJFC-S1800, living culture, SM28); *ibid*. (BJFC-S1801, living culture, CFCC 54351 = SM29); *ibid*. (BJFC-S1802, living culture, SM30); *ibid*. (BJFC-S1803, living culture, SM31); Hunan Province, Xiangtan City, 27°48′51″ N, 112°71′42″ E, alt. 85 m, on leaf spots of *Castanea mollissima*, 9 November 2020, C.M. Tian & N. Jiang (living culture, CFCC 54578 = XT01).

### 3.16. Coniella *Höhn*

**Figure 26:**
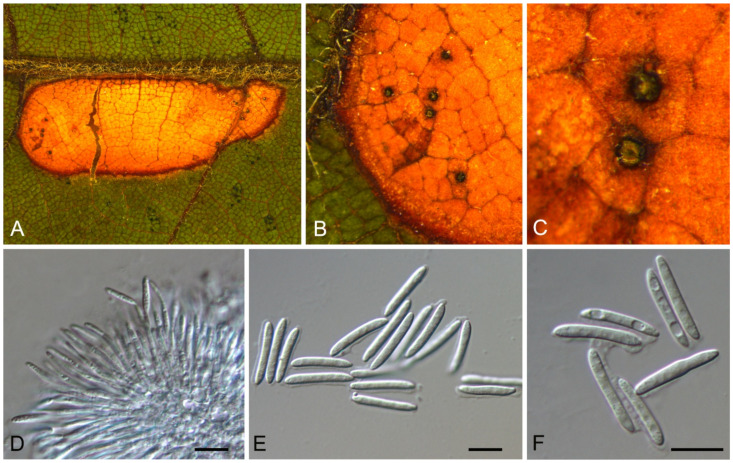
*Coniella castaneicola* (BJFC-S1793). (**A**–**C**) Conidiomata on the diseased leaves of *Castanea mollissima*; (**D**) Conidiogenous cells giving rise to conidia; (**E**,**F**) Conidia. Scale bars: D–F = 10 μm.

*Coniella castaneicola* B. Sutton, The Coelomycetes (Kew): 420 (1980) (Figure 26)

Pathogenic on *Castanea mollissima* leaves. Sexual morph: Undetermined. Asexual morph: Conidiomata 100–150 μm diam., pycnidial, conspicuous, scattered, globose to subglobose, black, single ostiolate. Conidiophores reduced to conidiogenous cells. Conidiogenous cells 4–13.5 × 1.5–3.5 μm, simple, hyaline, smooth, tapering. Conidia (16.7–)18.4–21.3(–22.3) × (2.7–)2.8–3.2(–3.3) μm ($\bar{x}$ = 19.9 × 3 μm, *n* = 50), L/W = (5.1–)6–7.2(–7.3) ($\bar{x}$ = 6.6, *n* = 50), aseptate, initially hyaline, becoming pale brown, smooth, cylindrical, linear, apex acute to nearly rounded, base truncate, smooth-walled, multiguttulate, enclosed in a persistent mucoid sheath.

Culture characteristics—Colonies on PDA reaching up to 60 mm in 10 days, flat, white, aerial mycelium spreads in irregular concentric zones. Conidiomata were formed after 15 days.

Material examined—CHINA, Guizhou Province, Zunyi City, Goujiang Town, 27°24′49″ N, 106°52′49″ E, alt. 1064 m, on leaf spots of *Castanea mollissima*, 7 September 2020, N. Jiang (BJFC-S1793, living culture, CFCC 54344 = ZY7-1); *ibid*. (living culture, ZY7-2).

**Figure A11:**
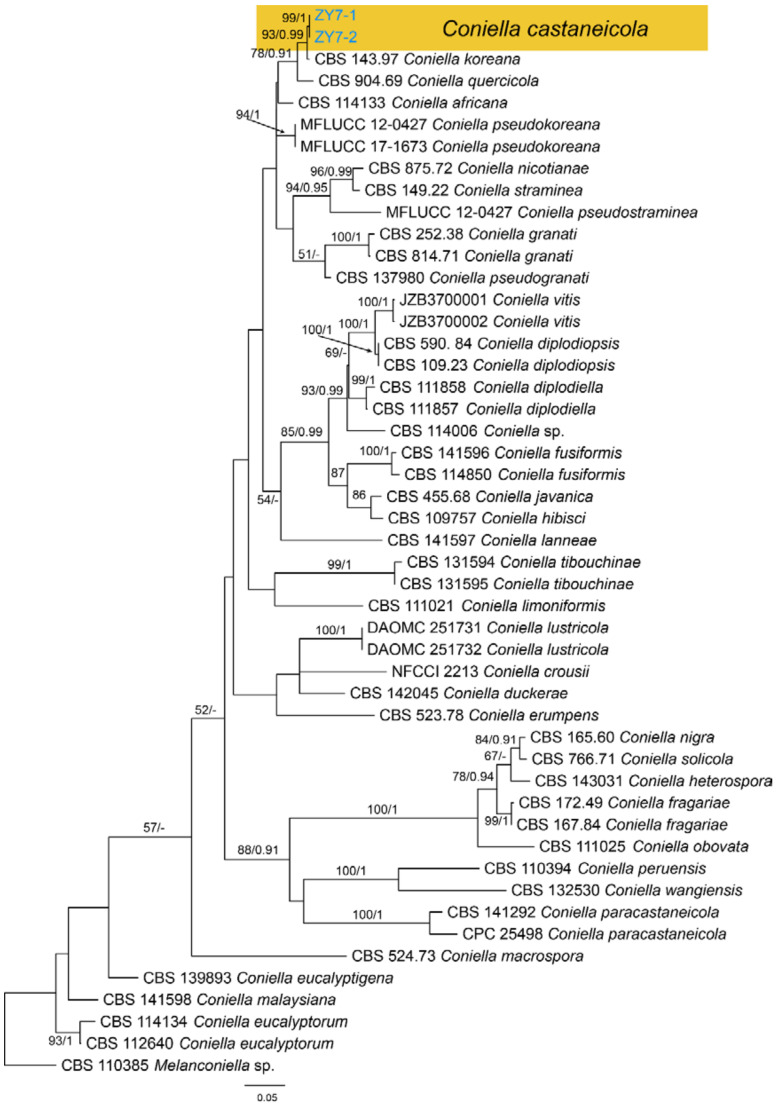
Phylogram generated from RAxML analysis based on combined ITS, *tef1* and *tub2* sequence data of *Coniella* isolates. The tree was rooted to *Melanconiella* sp. (CBS 110385). The scale bar indicates 0.05 nucleotide changes per site. Isolates from this study are marked in blue, and the identified species is marked in yellow.

Notes—The genus *Coniella* was well classified recently [60]. However, *Coniella castaneicola* was not studied for lacking of fresh collections and DNA data. In present study, we obtained fresh *Coniella* isolates from *Castanea mollissima*, and found it distinct from others in the phylogram (Figure A11). However, specimen in present study shared similar conidial morphology with the original description of *Coniella castaneicola* (20 × 2–2.5 μm) [61], hence we temporarily assign it to *C. castaneicola*.

### 3.17. Tubakia *B. Sutton*

**Figure 27:**
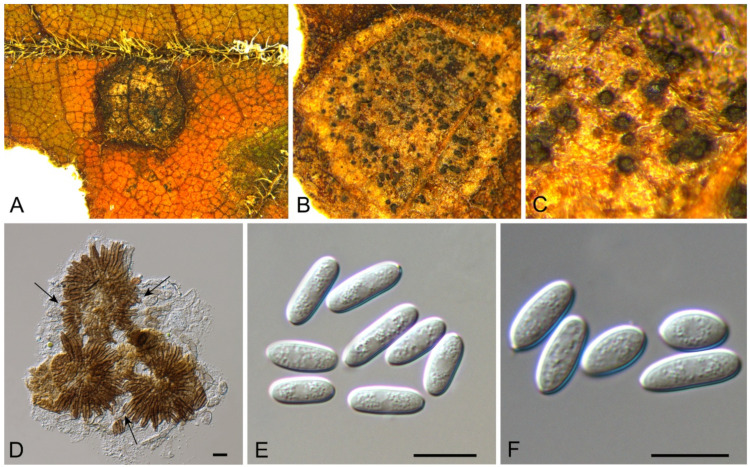
*Tubakia dryinoides* (BJFC-S1796). (**A**–**C**) Pycnothyria on the diseased leaves of *Castanea mollissima*; (**D**) Conidiogenous cells giving rise to conidia; (**E**,**F**) Conidia. Scale bars: D–F = 10 μm.

*Tubakia dryinoides* C. Nakash., Fungal Systematics and Evolution 1: 80 (2018) (Figure 27)

Pathogenic or saprobic on *Castanea mollissima* leaves. Sexual morph: Undetermined. Asexual morph: Conidiomata 70–120 μm diam., pycnothyrial, conspicuous, aggregated, superficial, circular or subcircular, black. Conidiophores reduced to conidiogenous cells, arising from the underside of the scutella, around the columella, radiating. Conidiogenous cells 5–14 × 2.5–5 μm, cylindrical, conical, hyaline to brown, thin-walled, smooth, apex obtuse to truncate. Conidia (10.4–)11.5–15.6(–17.7) × (5.1–)5.3–6.1(–6.5) μm ($\bar{x}\;$= 13.6 × 5.7 μm, *n* = 50), L/W = (1.7–)1.9–2.9(–3.4) ($\bar{x}\;$= 2.4, *n* = 50), aseptate, hyaline to pale brown, smooth, cylindrical to obovoid.

Culture characteristics—Colonies on PDA reaching up to 60 mm in 14 days, flat, creamy white, aerial mycelium forming concentric rings. Conidiomata were formed after 15 days.

Material examined—CHINA, Sichuan Province, Yaan City, Shimian County, 29°13′31″ N, 102°21′27″ E, alt. 978 m, on leaf spots of *Castanea mollissima*, 10 September 2020, N. Jiang (BJFC-S1796; living culture, CFCC 54346 = SM10-1); *ibid*. (living culture, = SM10).

**Figure A12:**
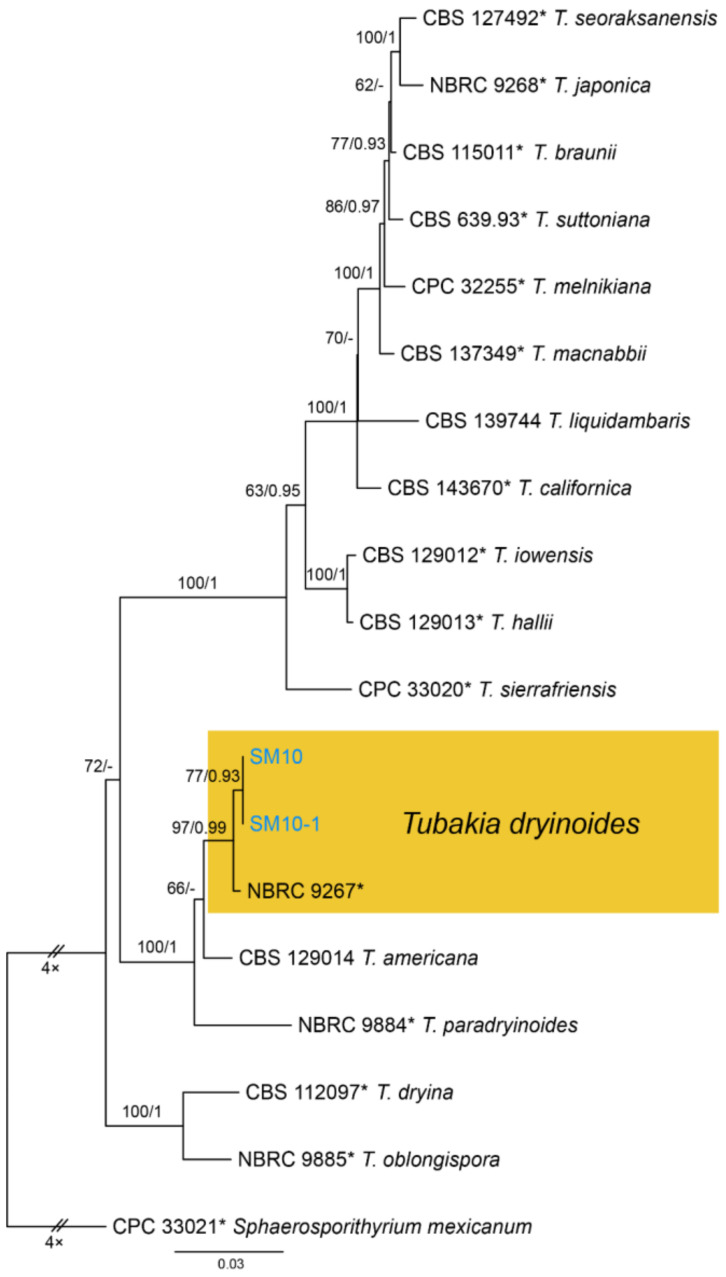
Phylogram generated from RAxML analysis based on combined ITS, *tef1* and *tub2* sequence data of *Tubakia* isolates. The tree was rooted to *Sphaerosporithyrium mexicanum* (CPC 33021). The scale bar indicates 0.03 nucleotide changes per site. Isolates from this study are marked in blue, ex-type strains are marked with *, and the identified species is marked in yellow.

Notes—*Tubakia dryinoides* was described from *Castanea crenata* and *Quercus phillyraeoides* in Japan [62]. In present study, strains from diseased chestnut leaves formed a supported clade with the ex-type strain NBRC 9267 (Figure A12), and shared similar morphology. Hence, we identified our strains as *T. dryinoides*, which represented a new host and geographical record.

### 3.18. Colletotrichum *Corda*

#### 3.18.1. *Colletotrichum fructicola*

*Colletotrichum fructicola* Prihast., L. Cai & K.D. Hyde, Fungal Divers. 39: 96 (2009)

Pathogenic on *Castanea henryi* and *C. mollissima* leaves. Sexual morph: Undetermined. Asexual morph was observed on PDA: Conidiomata, acervular, aggregated, orange. Conidiophores hyaline, septate, branched. Conidiogenous cells 5–17.5 × 1.5–3 μm, hyaline, cylindrical to ampulliform. Conidia (10.8–)11.2–16.9(–17.9) × (3.1–)3.2–5.4(–5.9) μm ($\bar{x}$ = 14.7 × 4.3 μm, *n* = 50), L/W = (3.1–)3.2–3.5(–3.6) ($\bar{x}$ = 3.4, *n* = 50), aseptate, hyaline, smooth, cylindrical, both ends rounded.

Culture characteristics—Colonies on PDA reaching up to 60 mm in 5 days, flat with entire edge, aerial mycelium dense, cottony, grey to dark grey in the centre, white at the margin; reverse greyish green.

Material examined—CHINA, Sichuan Province, Yaan City, Shimian County, 29°13′31″ N, 102°21′27″ E, alt. 978 m, on leaf spots of *Castanea mollissima*, 10 September 2020, N. Jiang (living cultures, SM6, SM9, SM13, SM14, SM14, SM16, SM30, SM31); Hunan Province, Shaoshan City, Yintian Town, 27°52′04″ N, 112°35′03″ E, alt. 73 m, on leaf spots of *Castanea mollissima*, 9 November 2020, C.M. Tian & N. Jiang (living cultures, SS01, SS14); Hunan Province, Xiangtan City, 27°48′51″ N, 112°71′42″ E, alt. 85 m, on leaf spots of *Castanea mollissima*, 9 November 2020, N. Jiang (living cultures, XT08, XT12, XT14-2, XT15); Hunan Province, Changsha City, Changsha County, Jinjing Town, 28°58′52″ N, 113°34′38″ E, alt. 63 m, on leaf spots of *Castanea henryi*, 10 November 2020, C.M. Tian & N. Jiang (living culture, JJ21).

**Figure A13:**
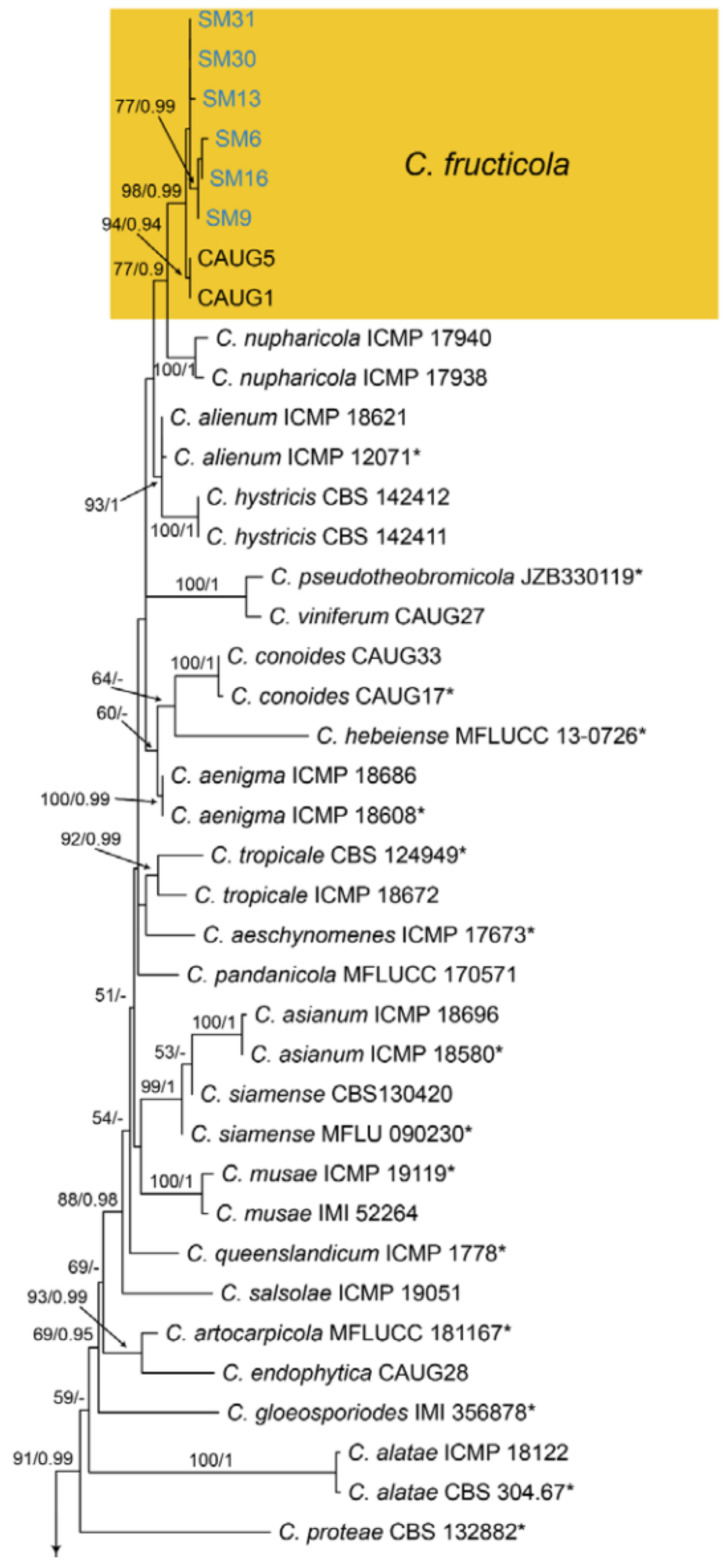

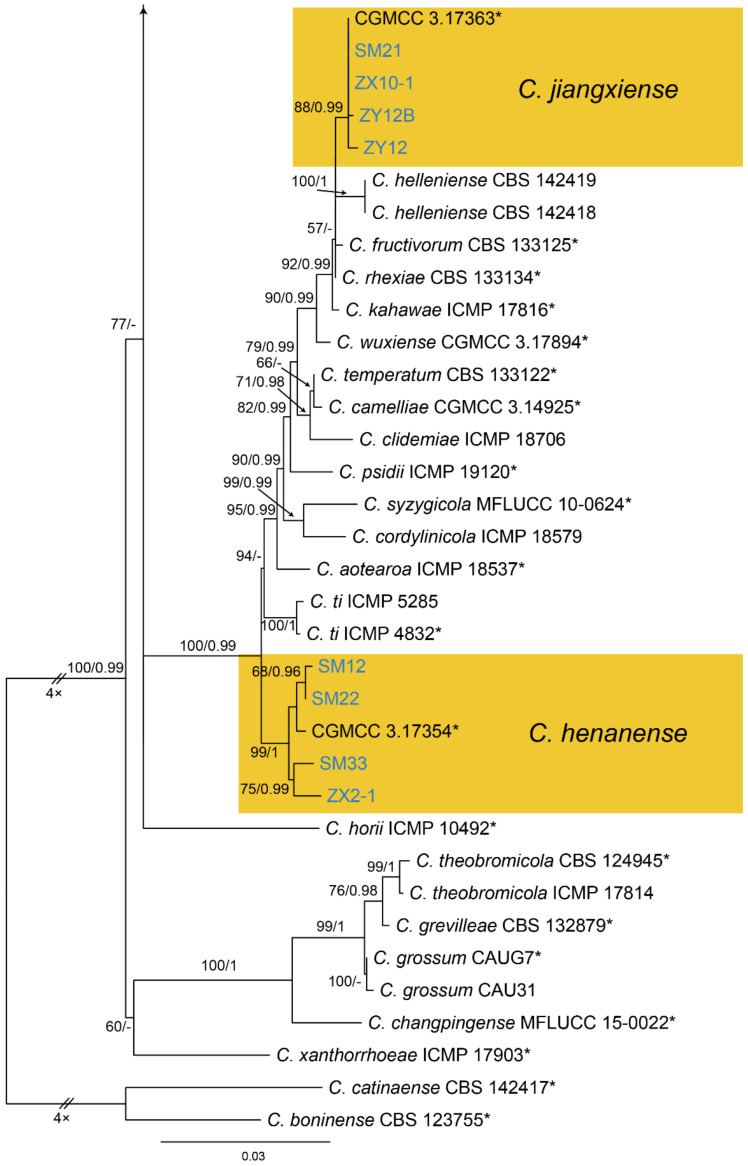
Phylogram generated from RAxML analysis based on combined ITS, *act*, *chs-1*, *gapdh* and *tub2* sequence data of *Collectrichum gloeosporiodes* species complex isolates. The tree was rooted to *Collectrichum catinaense* (CBS 142417) and *C. boninense* (CBS 123755). The scale bar indicates 0.03 nucleotide changes per site. Isolates from this study are marked in blue, ex-type strains are marked with *, and the identified species are marked in yellow.

Notes—*Colletotrichum fructicola* was described from *Coffea arabica* in Thailand [63], and subsequently found to infect several economic plants in China, such as *Camellia sinensis*, *Citrus sinensis*, *Morus alba*, *Pyrus pyrifolia* and *Vitis vinifera* [64]. In present study, strains from diseased chestnut leaves formed a supported clade with *Colletotrichum fructicola* (Figure A13), and shared similar morphology. Hence, we identified our strains as *C. fructicola*, and *Castanea henryi* and *C. mollissima* represented two new host records.

#### 3.18.2. *Colletotrichum henanense*

*Colletotrichum henanense* F. Liu & L. Cai, Persoonia 35: 80 (2015)

Pathogenic on *Castanea mollissima* leaves. Sexual morph: Undetermined. Asexual morph was observed on PDA: Conidiomata, acervular, aggregated, orange. Conidiophores hyaline, septate, branched. Conidiogenous cells 4.5–15 × 1.5–2.5 μm, hyaline, cylindrical to ampulliform. Conidia (8.8–)11.5–13.2(–17.2) × (3–)3.4–5.1(–5.8) μm ($\bar{x}\;$= 12.2 × 4.3 μm, *n* = 50), L/W = (2.5–)2.6–2.9(–3) ($\bar{x}\;$= 2.7, *n* = 50), aseptate, hyaline, smooth, cylindrical, both ends rounded.

Culture characteristics—Colonies on PDA reaching up to 60 mm in 5 days, flat with entire edge, aerial mycelium dense, cottony, grey to dark grey in the centre, white at the margin; reverse greyish green.

Material examined—CHINA, Sichuan Province, Yaan City, Shimian County, 29°13′31″ N, 102°21′27″ E, alt. 978 m, on leaf spots of *Castanea mollissima*, 10 September 2020, N. Jiang (living cultures, SM12, SM22, SM33); Hunan Province, Shaoshan City, Yintian Town, 27°52′04″ N, 112°35′03″ E, alt. 73 m, on leaf spots of *Castanea mollissima*, 9 November 2020, C.M. Tian & N. Jiang (living cultures, SS02, SS04); Yunnan Province, Zhaotong City, Zhenxiong County, 27°43′28″ N, 105°10′35″ E, alt. 1280 m, on leaf spots of *Castanea mollissima*, 5 September 2020, N. Jiang (living culture, ZX2-1); Hunan Province, Changsha City, Changsha County, Kaihui Town, 28°58′12″ N, 113°25′48″ E, alt. 65 m, on leaf spots of *Castanea mollissima*, 10 November 2020, C.M. Tian & N. Jiang (living culture, KH1).

Notes—*Colletotrichum henanense* was initially proposed as the leaf pathogen of *Camellia sinensis* and *Cirsium japonicum* in China [65]. Later, it was recorded to cause anthracnose of *Camellia oleifera* in China [66]. In the present study, strains from diseased chestnut leaves formed a supported clade with *Colletotrichum henanense* (Figure A13), and shared similar morphology. Hence, we identified our strains as *C. henanense*, and *Castanea mollissima* represented a new host record.

#### 3.18.3. *Colletotrichum jiangxiense*

*Colletotrichum jiangxiense* F. Liu & L. Cai, Persoonia 35: 82 (2015)

Pathogenic on *Castanea mollissima* leaves. Sexual morph: Undetermined. Asexual morph: Undetermined.

Culture characteristics—Colonies on PDA reaching up to 60 mm in 7 days, flat with entire edge, aerial mycelium dense, cottony, white to grey; reverse olivaceous.

Material examined—CHINA, Sichuan Province, Yaan City, Shimian County, 29°13′31″ N, 102°21′27″ E, alt. 978 m, on leaf spots of *Castanea mollissima*, 10 September 2020, N. Jiang (living culture, SM21); Yunnan Province, Zhaotong City, Zhenxiong County, 27°43′28″ N, 105°10′35″ E, alt. 1280 m, on leaf spots of *Castanea mollissima*, 5 September 2020, N. Jiang (living culture, ZX10-1); Guizhou Province, Zunyi City, Goujiang Town, 27°24′49″ N, 106°52′49″ E, alt. 1064 m, on leaf spots of *Castanea mollissima*, 7 September 2020, N. Jiang (living cultures, ZY12, ZY12B).

Notes—*Colletotrichum jiangxiense* was previously described from *Camellia sinensis* in China [65], and subsequently discovered from *Citrus sinensis* [67]. In present study, strains from diseased chestnut leaves formed a supported clade with *Colletotrichum jiangxiense* (Figure A13). Hence, we identified our strains as *C. jiangxiense*, and *Castanea mollissima* represented a new host record.

#### 3.18.4. *Colletotrichum karsti* You L. Yang, Zuo Y. Liu, K.D. Hyde & L. Cai, Cryptog. Mycol. 32(3): 241 (2011)

Pathogenic on *Castanea mollissima* leaves. Sexual morph: Undetermined. Asexual morph: Undetermined.

Culture characteristics—Colonies on PDA reaching up to 60 mm in 6 days, flat with entire margin, aerial mycelium dense, cottony, initially white, becoming grey with age; reverse pale brown.

Material examined—CHINA, Guizhou Province, Zunyi City, Goujiang Town, 27°24′49″ N, 106°52′49″ E, alt. 1064 m, on leaf spots of *Castanea mollissima*, 7 September 2020, N. Jiang (living cultures, CFCC 54365 = ZY3B, ZY3B-1).

**Figure A14:**
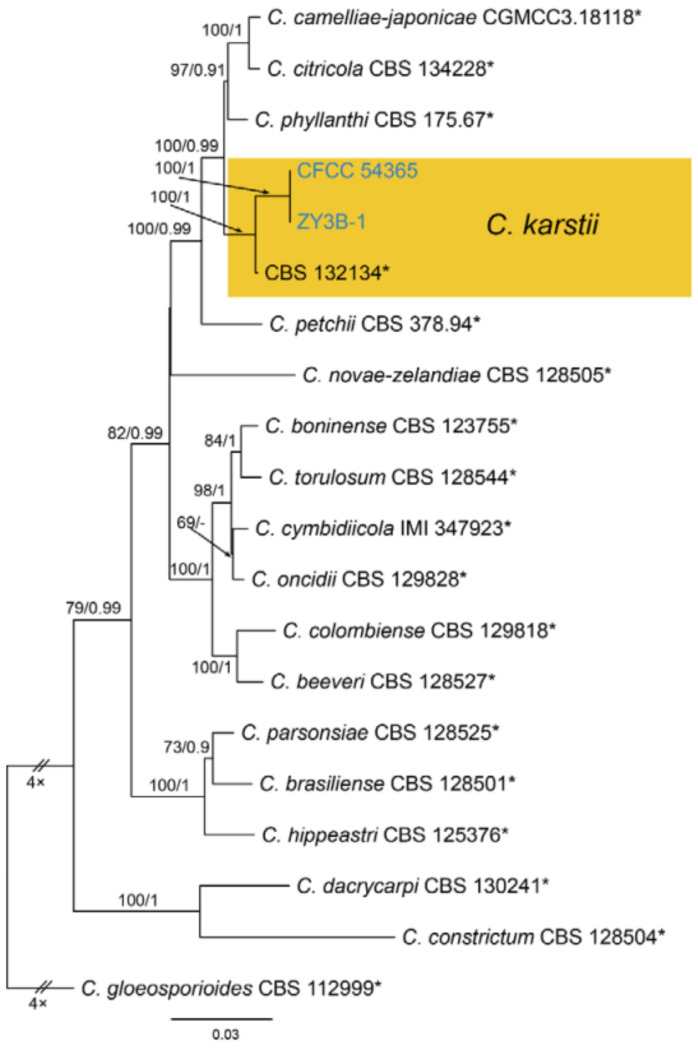
Phylogram generated from RAxML analysis based on combined ITS, *act*, *chs-1*, *gapdh* and *tub2* sequence data of *Collectrichum boninense* species complex isolates. The tree was rooted to *Collectrichum gloeosporiodes* (CBS 112999). The scale bar indicates 0.03 nucleotide changes per site. Isolates from this study are marked in blue, ex-type strains are marked with *, and the identified species is marked in yellow.

Notes—*Colletotrichum karsti* was described from *Arundina graminifolia*, *Calanthe argenteostriata*, *Eria coronaria*, *Pleione bulbocodioides* and *Vanda* sp. in China [68]. In the present study, strains from diseased chestnut leaves formed a supported clade with *Colletotrichum karsti* (Figure A14). Hence, we identified our strains as *C. karsti*, and *Castanea mollissima* represented a new host record.

#### 3.18.5. *Colletotrichum nymphaeae* (Pass.) Aa, Netherlands Journal of Plant Pathology, Supplement 1 84(3): 110 (1978)

Pathogenic on *Castanea mollissima* leaves. Sexual morph: Undetermined. Asexual morph: Undetermined.

Culture characteristics—Colonies on PDA reaching up to 60 mm in 8 days, flat with entire edge, aerial mycelium dense, cottony, white to grey; reverse olivaceous.

Material examined—CHINA, Sichuan Province, Yaan City, Shimian County, 29°13′31″ N, 102°21′27″ E, alt. 978 m, on leaf spots of *Castanea mollissima*, 10 September 2020, N. Jiang (living cultures, CFCC 54366 = SM26, SM26-1).

**Figure A15:**
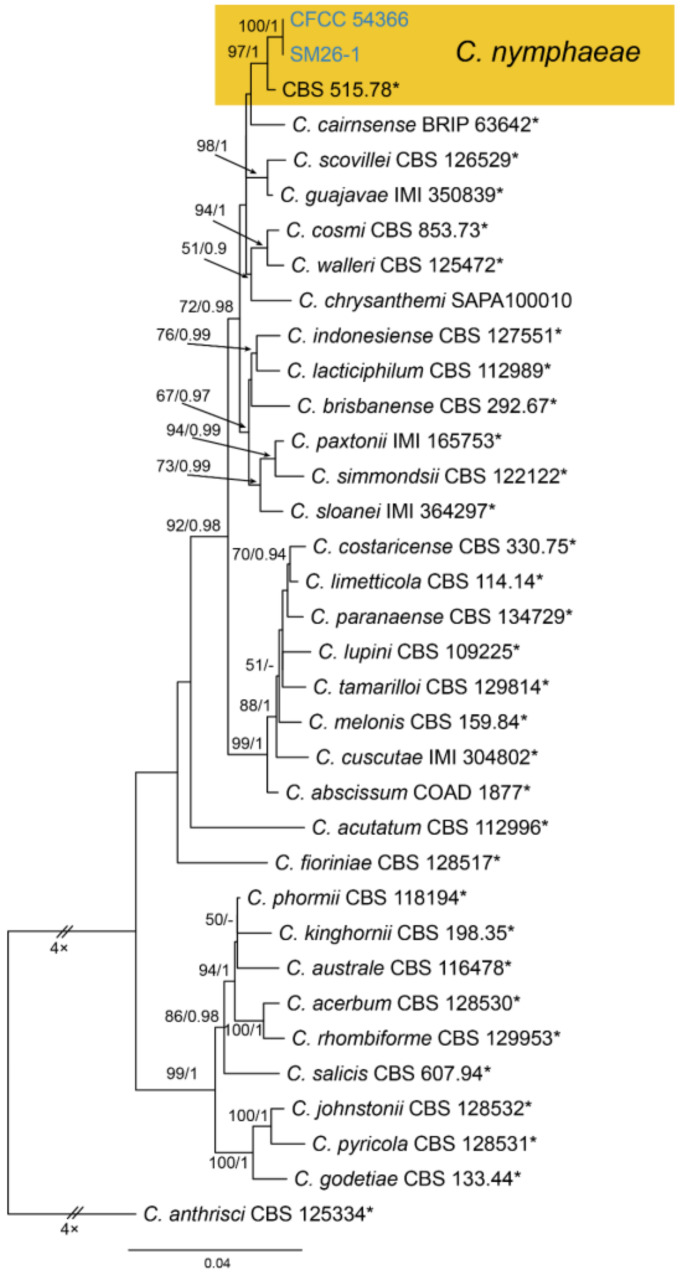
Phylogram generated from RAxML analysis based on combined ITS, *act*, *chs-1*, *gapdh* and *tub2* sequence data of *Collectrichum acutatum* species complex isolates. The tree was rooted to *Collectrichum anthrisci* (CBS 125334). The scale bar indicates 0.04 nucleotide changes per site. Isolates from this study are marked in blue, ex-type strains are marked with *, and the identified species is marked in yellow.

Notes—*Colletotrichum nymphaeae* was recorded to be associated with several hosts, including *Camellia oleifera*, *Citrus aurantifolia*, *Juglans regia*, *Malus domestica*, *Prunus salicina* and *Vitis vinifera* [69]. In the present study, strains from diseased chestnut leaves formed a supported clade with *Colletotrichum nymphaeae* (Figure A15). Hence, we identified our strains as *C. nymphaeae*, and *Castanea mollissima* represented a new host record.

## 4. Discussion

*Castanea henryi* and *C. mollissima* are two crops currently cultivated in plantations of China, and suffering from cankers, leaf spots and fruit rot diseases commonly. During our investigations in the past years, *Cryphonectria parasitica* and *Dendrostoma* spp. were commonly occurring in most plantations, causing mild to serious cankers depending on the management [2,10]. *Gnomoniopsis chinensis* caused fatal stem and branch canker disease in only Hebei Province [60]. Compared to the cankers, leaf spots are usually neglected. In present study, we focused on the leaf-inhabiting fungi, identified them to 26 fungal species using phenotypic characters and the multi-locus phylogeny.

From Table 3, most fungi (92.3%) belong to Sordariomycetes and the rest two species belong to Dothideomycetes. This result is nearly congruent as we expected, because Sordariomycetes is a species-rich class and contains many plant pathogens [69]. Within Sordariomycetes, Amphisphaeriales (45.8%), Botryosphaeriales (12.5%), Diaporthales (33.3%) and Glomerellales (20.8%) are identified. They contain famous plant pathogens such as pestalotioid taxa, Botryosphaeria-like taxa, diaporthalean fungi and *Colletotrichum* species. These fungi were documented in the monograph of chestnut disease by Xie in 1998 [44]. However, genus and species concepts have changed a lot in recent years. For example, the old name *Colletotrichum gloeosporioides* has been expanded to a group of species named *Colletotrichum gloeosporioides* species complex [70], hence the chestnut-inhabiting *Colletotrichum* needs to be re-identified to particular one or several species. The genus *Pestalotiopsis s. l.* was separated into three genera, namely *Pestalotiopsis s. s.*, *Neopestalotiopsis* and *Pseudopestalotiopsis* based on phylogeny [50]. In addition, species with similar morphology from the same host, especially resulting into same symptoms, are not easy to be distinguished without molecular approach in previous studies. *Gnomoniopsis daii* from Chinese chestnut and *Gnomoniopsis smithogilvyi* (*G. castaneae*) from European chestnut were likely identified to *Phomopsis* (now *Diaporthe*) species for extremely similar morphology.

According to the filed investigation and sample observation, *Colletotrichum* spp., *Neopestalotiopsis* spp., *Pestalotiopsis kenyana* and *Phyllosticta capitalensis* are now common pathogens in plantations of *Castanea henryi* and *C. mollissima*. Pathogenicity tests and disease control methods are required to be conducted in the future.

## Figures and Tables

**Table 1:** Selected genes and primers for polymerase chain reaction of each genus.

Genera	ITS [16]	LSU [16]	*act* [17]	*cal* [17]	*chs-1* [17]	*gapdh* [18,19]	*his3* [20,21]	*rpb2* [22]	*tef1* [17]	*tub2* [20]
*Aplosporella*	ITS1/ITS4								EF1-688F/EF1-1251R	
*Arthrinium*	ITS1/ITS4									
*Aureobasidium*	ITS1/ITS4	LR0R/LR5								
*Bartalinia pini*	ITS1/ITS4	LR0R/LR5								
*Botryosphaeria*	ITS1/ITS4								EF1-688F/EF1-1251R	TI/BT2B
*Colletotrichum*	ITS1/ITS4		ACT-512F/ACT-783R		CHS-79F/CHS-345R	GDF/GDR				TI/BT2B
*Coniella*	ITS1/ITS4								EF1-688F/EF1-1251R	
*Diaporthe*	ITS1/ITS4			CAL-228F/CAL-737R			CYLH4F/H3-1b		EF1-688F/EF1-1251R	TI/BT2B
*Didymella*	ITS1/ITS4	LR0R/LR5						RPB2-5F2/RPB2-7CR		TI/BT2B
*Discosia*	ITS1/ITS4	LR0R/LR5								TI/BT2B
*Gnomoniopsis*	ITS1/ITS4								EF1-688F/EF1-1251R	TI/BT2B
*Monochaetia*	ITS1/ITS4	LR0R/LR5						RPB2-5F2/RPB2-7CR	EF1-688F/EF1-1252R	TI/BT2B
*Neopestalotiopsis*	ITS1/ITS4								EF1-688F/EF1-1253R	TI/BT2B
*Pestalotiopsis*	ITS1/ITS4								EF1-688F/EF1-1254R	TI/BT2B
*Phyllosticta*	ITS1/ITS4		ACT-512F/ACT-783R			Gpd1-LM/Gpd2-LM				
*Pyrispora*	ITS1/ITS4	LR0R/LR5						RPB2-5F2/RPB2-7CR	EF1-688F/EF1-1254R	
*Robillarda*	ITS1/ITS4	LR0R/LR5						RPB2-5F2/RPB2-7CR	EF1-688F/EF1-1255R	TI/BT2B
*Tubakia*	ITS1/ITS4								EF1-688F/EF1-1256R	TI/BT2B

**Table 2:** Isolates and GenBank accession numbers of sequences from this study.

Species	Isolates	GenBank Accession No.									
		ITS	LSU	*act*	*cal*	*chs-1*	*gapdh*	*his3*	*rpb2*	*tef1*	*tub2*
*Aplosporella prunicola*	CFCC 54334 = SM18B	MW350059	NA	NA	NA	NA	NA	NA	NA	MW381858	NA
*Aplosporella prunicola*	SM18B-1	MW350060	NA	NA	NA	NA	NA	NA	NA	MW381859	NA
*Arthrinium arundinis*	XT18-1	MW364286	NA	NA	NA	NA	NA	NA	NA	NA	NA
*Aureobasidium castaneae* sp. nov.	CFCC 54591 = JJ7-3	MW364284	MW364275	NA	NA	NA	NA	NA	NA	NA	NA
*Bartalinia pini*	CFCC 54574 = JJ4	MW364285	MW364276	NA	NA	NA	NA	NA	NA	NA	NA
*Botryosphaeria dothidea*	JJ2B	MW350061	NA	NA	NA	NA	NA	NA	NA	MW381860	MW381864
*Botryosphaeria dothidea*	CFCC 54576 = JJ12	MW350062	NA	NA	NA	NA	NA	NA	NA	MW381861	MW381865
*Botryosphaeria dothidea*	JJ14	MW350063	NA	NA	NA	NA	NA	NA	NA	MW381862	MW381866
*Botryosphaeria dothidea*	JJ27-1	MW350064	NA	NA	NA	NA	NA	NA	NA	MW381863	MW381867
*Colletotrichum fructicola*	SM6	MW217249	NA	MW227352	NA	MW227370	MW381824	NA	NA	NA	MW227388
*Colletotrichum fructicola*	SM9	MW217250	NA	MW227353	NA	MW227371	MW381825	NA	NA	NA	MW227389
*Colletotrichum fructicola*	CFCC 54363 = SM13	MW217251	NA	MW227354	NA	MW227372	MW381826	NA	NA	NA	MW227390
*Colletotrichum fructicola*	SM16	MW217252	NA	MW227355	NA	MW227373	MW381827	NA	NA	NA	MW227391
*Colletotrichum fructicola*	SM30	MW217253	NA	MW227356	NA	MW227374	MW381828	NA	NA	NA	MW227392
*Colletotrichum fructicola*	SM31	MW217254	NA	MW227357	NA	MW227375	MW381829	NA	NA	NA	MW227393
*Colletotrichum henanense*	CFCC 54364 = SM12	MW217255	NA	MW227358	NA	MW227376	MW381830	NA	NA	NA	MW227394
*Colletotrichum henanense*	SM22	MW217256	NA	MW227359	NA	MW227377	MW381831	NA	NA	NA	MW227395
*Colletotrichum henanense*	SM33	MW217257	NA	MW227360	NA	MW227378	MW381832	NA	NA	NA	MW227396
*Colletotrichum henanense*	ZX2-1	MW217258	NA	MW227361	NA	MW227379	MW381833	NA	NA	NA	MW227397
*Colletotrichum jiangxiense*	SM21	MW217259	NA	MW227362	NA	MW227380	MW381834	NA	NA	NA	MW227398
*Colletotrichum jiangxiense*	CFCC 54362 = ZX10-1	MW217260	NA	MW227363	NA	MW227381	MW381835	NA	NA	NA	MW227399
*Colletotrichum jiangxiense*	ZY12B	MW217261	NA	MW227364	NA	MW227382	MW381836	NA	NA	NA	MW227400
*Colletotrichum jiangxiense*	ZY12	MW217262	NA	MW227365	NA	MW227383	MW381837	NA	NA	NA	MW227401
*Colletotrichum karsti*	CFCC 54365 = ZY3B	MW217263	NA	MW227366	NA	MW227384	MW381838	NA	NA	NA	NA
*Colletotrichum karsti*	ZY3B-1	MW217264	NA	MW227367	NA	MW227385	MW381839	NA	NA	NA	NA
*Colletotrichum nymphaeae*	CFCC 54366 = SM26	MW217265	NA	MW227368	NA	MW227386	MW381840	NA	NA	NA	MW227402
*Colletotrichum nymphaeae*	SM26-1	MW217266	NA	MW227369	NA	MW227387	MW381841	NA	NA	NA	MW227403
*Coniella castaneicola*	CFCC 54344 = ZY7-1	MW208111	NA	NA	NA	NA	NA	NA	NA	MW227343	NA
*Coniella castaneicola*	ZY7-2	MW208112	NA	NA	NA	NA	NA	NA	NA	MW227344	NA
*Diaporthe lithocarpi*	CFCC 54573 = JJ3	MW364281	NA	NA	MW381842	NA	NA	MW381845	NA	MW381848	MW381851
*Diaporthe lithocarpi*	JJ3-2	MW364282	NA	NA	MW381843	NA	NA	MW381846	NA	MW381849	MW381852
*Diaporthe lithocarpi*	JJ26B	MW364283	NA	NA	MW381844	NA	NA	MW381847	NA	MW381850	MW381853
*Didymella coffeae-arabicae*	CFCC 54343 = SM24	MW364357	MW364277	NA	NA	NA	NA	NA	MW381854	NA	MW381856
*Didymella coffeae-arabicae*	SM24B	MW364358	MW364278	NA	NA	NA	NA	NA	MW381855	NA	MW381857
*Discosia castaneae* sp. nov.	CFCC 54088 = CML1	MN842798	MN842796	NA	NA	NA	NA	NA	NA		MN864778
*Discosia castaneae* sp. nov.	CML2	MN842799	MN842797	NA	NA	NA	NA	NA	NA		MN864779
*Gnomoniopsis daii*	CFCC 54345 = ZY11	MW208113	NA	NA	NA	NA	NA	NA	NA	MW227345	MW218543
*Gnomoniopsis daii*	ZY10-1	MW208114	NA	NA	NA	NA	NA	NA	NA	MW227346	MW218544
*Gnomoniopsis daii*	ZY10-3	MW208115	NA	NA	NA	NA	NA	NA	NA	MW227347	MW218545
*Gnomoniopsis daii*	ZY12A	MW208116	NA	NA	NA	NA	NA	NA	NA	MW227348	MW218546
*Gnomoniopsis daii*	ZX14-1	MW208117	NA	NA	NA	NA	NA	NA	NA	MW227349	MW218547
*Monochaetia castaneae* sp. nov.	CFCC 54354 = SM9-1	MW166222	MW166263	NA	NA	NA	NA	NA	MW199737	MW199741	MW218515
*Monochaetia castaneae* sp. nov.	SM9-2	MW166223	MW166264	NA	NA	NA	NA	NA	MW199738	MW199742	MW218516
*Neopestalotiopsis asiatica*	CFCC 54339 = SM32	MW166224	NA	NA	NA	NA	NA	NA	NA	MW199743	MW218517
*Neopestalotiopsis asiatica*	SM7	MW166225	NA	NA	NA	NA	NA	NA	NA	MW199744	MW218518
*Neopestalotiopsis asiatica*	SM8B	MW166226	NA	NA	NA	NA	NA	NA	NA	MW199745	MW218519
*Neopestalotiopsis asiatica*	SM11	MW166227	NA	NA	NA	NA	NA	NA	NA	MW199746	MW218520
*Neopestalotiopsis asiatica*	SM19B	MW166228	NA	NA	NA	NA	NA	NA	NA	MW199747	MW218521
*Neopestalotiopsis brasiliensis*	CFCC 54341 = ZY4	MW166229	NA	NA	NA	NA	NA	NA	NA	MW199748	MW218522
*Neopestalotiopsis brasiliensis*	ZY4-2D	MW166230	NA	NA	NA	NA	NA	NA	NA	MW199749	MW218523
*Neopestalotiopsis sichuanensis* sp. nov.	CFCC 54338 = SM15-1	MW166231	NA	NA	NA	NA	NA	NA	NA	MW199750	MW218524
*Neopestalotiopsis sichuanensis* sp. nov.	SM15-1C	MW166232	NA	NA	NA	NA	NA	NA	NA	MW199751	MW218525
*Neopestalotiopsis* sp.1	CFCC 54337 = ZX12A	MW166233	NA	NA	NA	NA	NA	NA	NA	MW199752	MW218526
*Neopestalotiopsis* sp.1	ZX12-1	MW166234	NA	NA	NA	NA	NA	NA	NA	MW199753	MW218527
*Neopestalotiopsis* sp.2	CFCC 54340 = SM14	MW166235	NA	NA	NA	NA	NA	NA	NA	MW199754	MW218528
*Neopestalotiopsis* sp.2	ZX22B	MW166236	NA	NA	NA	NA	NA	NA	NA	MW199755	MW218529
*Pestalotiopsis kenyana*	CFCC 54336 = ZX11	MW166237	NA	NA	NA	NA	NA	NA	NA	MW199756	MW218530
*Pestalotiopsis kenyana*	ZX3	MW166238	NA	NA	NA	NA	NA	NA	NA	MW199757	MW218531
*Pestalotiopsis kenyana*	ZX7	MW166239	NA	NA	NA	NA	NA	NA	NA	MW199758	MW218532
*Pestalotiopsis kenyana*	ZX9	MW166240	NA	NA	NA	NA	NA	NA	NA	MW199759	MW218533
*Pestalotiopsis kenyana*	ZX18A	MW166241	NA	NA	NA	NA	NA	NA	NA	MW199760	MW218534
*Phyllosticta capitalensis*	CFCC 54577 = JJ16	MW350068	NA	MW381868	NA	NA	MW381879	NA	NA	NA	NA
*Phyllosticta capitalensis*	JJ20	MW350069	NA	MW381869	NA	NA	MW381880	NA	NA	NA	NA
*Phyllosticta capitalensis*	SS07	MW350070	NA	MW381870	NA	NA	MW381881	NA	NA	NA	NA
*Phyllosticta capitalensis*	SS10	MW350071	NA	NA	NA	NA	MW381882	NA	NA	NA	NA
*Phyllosticta capitalensis*	SS13	MW350072	NA	MW381871	NA	NA	MW381883	NA	NA	NA	NA
*Phyllosticta capitalensis*	SS15	MW350073	NA	MW381872	NA	NA	MW381884	NA	NA	NA	NA
*Phyllosticta capitalensis*	SS16-1	MW350074	NA	NA	NA	NA	MW381885	NA	NA	NA	NA
*Phyllosticta capitalensis*	SS16-2	MW350075	NA	NA	NA	NA	MW381886	NA	NA	NA	NA
*Phyllosticta capitalensis*	CFCC 54579 = XT10	MW350076	NA	MW381873	NA	NA	MW381887	NA	NA	NA	NA
*Phyllosticta capitalensis*	XT11	MW350077	NA	MW381874	NA	NA	MW381888	NA	NA	NA	NA
*Phyllosticta capitalensis*	XT16-1	MW350078	NA	MW381875	NA	NA	MW381889	NA	NA	NA	NA
*Phyllosticta capitalensis*	XT16-2	MW350079	NA	MW381876	NA	NA	MW381890	NA	NA	NA	NA
*Phyllosticta capitalensis*	XT17	MW350080	NA	MW381877	NA	NA	MW381891	NA	NA	NA	NA
*Phyllosticta capitalensis*	CFCC 54355 = ZX6	MW350081	NA	NA	NA	NA	MW381892	NA	NA	NA	NA
*Phyllosticta capitalensis*	ZX11-1	MW350082	NA	NA	NA	NA	MW381893	NA	NA	NA	NA
*Phyllosticta capitalensis*	CFCC 54356 = ZY6-1	MW350083	NA	MW381878	NA	NA	MW381894	NA	NA	NA	NA
*Pyrispora castaneae* sp. nov.	CFCC 54349 = SM17	MW208108	MW208105	NA	NA	NA	NA	NA	MW218535	MW227340	NA
*Pyrispora castaneae* sp. nov.	CFCC 54350 = SM20	MW208109	MW208106	NA	NA	NA	NA	NA	MW218536	MW227341	NA
*Pyrispora castaneae* sp. nov.	CFCC 54351 = SM29	MW208110	MW208107	NA	NA	NA	NA	NA	MW218537	MW227342	NA
*Robillarda sessilis*	CFCC 54353 = ZX5	MW166242	MW166265	NA	NA	NA	NA	NA	MW199739	MW218550	MW218553
*Robillarda sessilis*	ZX5-1	MW166243	MW166266	NA	NA	NA	NA	NA	MW199740	MW218551	MW218554
*Robillarda sessilis*	ZY5	MW218478	MW218479	NA	NA	NA	NA	NA	MW222613	MW218552	MW218555
*Tubakia dryinoides*	CFCC 54346 = SM10-1	MW208118	NA	NA	NA	NA	NA	NA	NA	MW227350	MW218548
*Tubakia dryinoides*	SM10	MW208119	NA	NA	NA	NA	NA	NA	NA	MW227351	MW218549

NA: Not available.

## Data Availability

All sequence data are available in NCBI GenBank following the accession numbers in the manuscript.
